# Latitudinal Clines in Gene Flow and Demographic Stability Reveal Drivers of Microendemism in a Radiation of Alpine Grasshoppers

**DOI:** 10.1111/mec.70332

**Published:** 2026-04-03

**Authors:** Joaquín Ortego, Marina Trillo, Jorge Gutiérrez‐Rodríguez, Vicente García‐Navas

**Affiliations:** ^1^ Department of Ecology and Evolution, Estación Biológica de Doñana, EBD‐CSIC Seville Spain; ^2^ Evolutionary Biology Program, Department of Ecology and Genetics (IEG), Uppsala University Uppsala Sweden

**Keywords:** alpine radiations, geometric morphometrics, microendemism, pleistocene speciation, range‐size evolution, reproductive isolation, speciation, species delimitation

## Abstract

Understanding the geological, ecological, and microevolutionary processes that shape range size in alpine organisms is key to explaining the high rates of local endemism that characterise mountain ecosystems. Here, we investigate the geography of speciation in *Oropodisma*, a radiation of alpine grasshoppers from the Balkan Peninsula that includes several narrow‐endemic species distributed along a latitudinal gradient of > 600 km. Phylogenomic‐based species delimitation clarified taxonomic uncertainties and revealed striking variation in range size, with the northernmost species occupying an area nearly as large as all other congeneric taxa combined. Phylogenetic network analyses and divergence dating indicate a Pleistocene origin for the group, with recent speciation events (< 0.2 Ma) and limited post‐divergence gene flow, suggesting rapid evolution of reproductive isolation and short speciation times. Distributional shifts inferred from environmental niche modelling show that all taxa underwent severe range contractions during interglacial periods, leading to population fragmentation and demographic declines. Despite the ubiquity of these processes across taxa, both demographic stability and genetic connectivity among conspecific populations decreased at lower latitudes relative to the genus' distribution. This suggests that greater genetic connectivity at higher relative latitudes, likely driven by regional range shifts and a more continuous availability of suitable habitats through time, has limited opportunities for microgeographic speciation and maintained genetic cohesiveness among populations across broader distributional ranges. Collectively, our findings support a model of interglacial speciation and illustrate how latitudinal variation in the balance between lineage formation and fusion—shaped by Pleistocene climatic oscillations—determines the rates at which alpine microendemic species emerge and accumulate in temperate mountain regions.

## 1 Introduction

Mountain ecosystems harbour a disproportionately high number of species compared to other regions on Earth, and their alpine habitats often include numerous narrow endemics with ranges restricted to one or a few mountain tops (Steinbauer et al. 2016; Rahbek et al. 2019; e.g., Noroozi et al. 2018). Yet, our understanding of the evolutionary, ecological, and geological processes underlying the origin of these micro‐endemic alpine species remains limited (Antonelli et al. 2018; Rahbek et al. 2019; Perrigo et al. 2020; e.g., Pallarés et al. 2024; Moreyra et al. 2025). In their landmark paper on the controls of speciation, Dynesius and Jansson (2014) argued that the rate at which new species arise depends not only on the frequency of initiation of within‐species lineages (i.e., rate of splitting) and the time required for the completion of reproductive isolation (i.e., speciation duration), but also on the persistence of diverging lineages before they became fully separated into distinct species. Deciphering the proximate processes determining these microevolutionary controls of speciation is thus essential to understanding the drivers of alpine diversity and the origins of the many microendemic species that characterise high mountain habitats. Despite this well‐established theoretical framework and its potential for understanding range size variation and the uneven accumulation of narrow endemic species across regions, empirical tests assessing how geographic context modulates splitting rates, lineage persistence, and the completion of speciation remain scarce (Dynesius and Jansson 2000). Adopting such a process‐based inference framework is also key for assessing the relative role of mountains as “cradles” and/or “museums” of biodiversity (Vasconcelos et al. 2022), metaphors that have been frequently invoked to distinguish regions of high species richness according to whether diversity results mainly from high rates of speciation (i.e., cradles of young, recently derived species) or from low rates of extinction (i.e., museums accumulating old, early‐divergent species) (e.g., Li et al. 2021; Sonne et al. 2022).

In mountain ecosystems, high topographic complexity and barriers to dispersal provide favourable conditions for the initiation of divergence during periods of range contraction and population isolation (Perrigo et al. 2020). This is reflected in the pronounced genetic structure documented in many montane and alpine taxa, resulting from disruption of gene flow among isolated or partially isolated populations (i.e., high rates of lineage formation) (e.g., Ægisdóttir et al. 2009; Noguerales et al. 2016; Osborne et al. 2019; Tonzo et al. 2019; Hartley et al. 2023). Mountains are also expected to foster lineage persistence through steep ecological gradients that allow species to track suitable habitats via elevational shifts during environmental change, thereby reducing extinction risk (Sandel et al. 2011; Steinbauer et al. 2013, 2016). These favourable conditions for divergence and persistence, however, were likely counterbalanced by Pleistocene glacial–interglacial cycles, which are thought to have exerted antagonistic effects on speciation. On the one hand, climatic oscillations undermined lineage persistence when secondary contact during range expansions led to lineage fusion and speciation reversal (Jansson and Dynesius 2002; Garrick et al. 2019; e.g., Maier et al. 2019); on the other hand, they promoted lineage formation during periods of range contraction and population isolation in glacial or interglacial refugia (Carstens and Knowles 2007; Nevado et al. 2018; Ortego and Knowles 2022). Speciation duration can ultimately tip the balance between these antagonistic processes, determining whether new lineages achieve complete or partial reproductive isolation rapidly enough to prevent speciation reversal during periods of secondary contact (Dynesius and Jansson 2014). Several studies have indeed reported short speciation times in montane and alpine organisms, suggesting that rapid evolution of reproductive isolation is crucial to prevent lineage fusion during range expansions, which in turn may explain the extraordinary diversity of some alpine biotas (e.g., Ortego et al. 2024; Wei et al. 2022; Voisin et al. 2025). Paradoxically, while rapid speciation may help explain why mountain regions are global biodiversity hotspots, it also poses a major challenge, as most alpine radiations involve young allopatric lineages that typically combine strong genetic divergence with only subtle phenotypic differentiation (Voisin et al. 2025; Moreyra et al. 2025; García‐Navas et al. 2025). Thus, integrating speciation‐based taxonomic delimitation, demographic inference, and modelling of distributional shifts and genetic connectivity across the landscape offers a comprehensive framework to assess the true diversity of mountain ecosystems, accurately delineate species ranges, and elucidate the processes underlying their origin and persistence through evolutionary time.

Considering the geographical dimension of speciation across latitudinal gradients provides a powerful framework for testing general predictions about how isolation, connectivity, and demographic stability shape splitting rates, lineage persistence, and, ultimately, range size variation and the formation of narrow endemic species. In low‐latitude ranges, long‐term population fragmentation may have enhanced opportunities for divergence but also increased extinction risk when suitable habitats contracted below viability thresholds during interglacial periods (Stewart et al. 2010). By contrast, higher‐latitude mountains likely offered greater continuity of suitable habitats, contributing to increased population connectivity through time and long‐term persistence but limiting opportunities for diversification. This reduced potential for microgeographic speciation is also likely exacerbated by faster climatic velocity at higher latitudes, which is expected to promote gene pool homogenisation through repeated range shifts driven by glacial–interglacial cycles (Dynesius and Jansson 2000). Under this framework, latitudinal gradients are likely to reflect predictable variation in the balance between lineage formation and lineage fusion. Thus, biodiversity of alpine systems from temperate regions is expected to mirror the contrasting demographic and evolutionary dynamics shaped by the heterogeneous impacts of climatic oscillations across latitudes. Lying at the transition between northern temperate regions heavily impacted by Pleistocene glaciations and lower‐latitude areas where these effects were far more limited, the Mediterranean mountains provide an exceptional biogeographical setting to investigate the processes driving the formation of narrow endemic alpine species and to address the ongoing debate on whether Pleistocene glacial cycles acted primarily as ignitors or inhibitors of speciation (Klicka and Zink 1997; Dynesius and Jansson 2000; Jansson and Dynesius 2002).

In this study, we employ an integrative, process‐based framework to investigate how Pleistocene climatic oscillations, demographic dynamics, and microevolutionary processes collectively shape diversification in alpine systems (Tzedakis 2009; Feliner 2014; Arroyo et al. 2022). To this end, we focus on the genus *Oropodisma* Uvarov, 1942, a radiation of alpine grasshoppers from the Balkans distributed across a broad latitudinal gradient (> 600 km), extending from the Sharr Mountains in North Macedonia and Kosovo to Mount Taygetus at the southernmost tip of continental Greece (Cigliano et al. 2025; Trillo and Ortego 2025). The genus currently comprises 12 valid species, which are morphologically very similar and were primarily described based on subtle differences in the shape of the male internal genitalia and furculae (Trillo and Ortego 2025 and references therein). All putative species are allopatric and restricted to the same elevational belt of open alpine and subalpine habitats (generally above 1800 m), with most taxa confined to a single mountain or a few adjacent ranges. Remarkably, the taxonomic status of some populations remains uncertain (Willemse and Willemse 2008; Trillo and Ortego 2025). The broad latitudinal gradient over which the genus is distributed makes it an ideal case study to test the context‐dependent role of Pleistocene glaciations in promoting or constraining speciation, and to investigate the mechanisms driving diversification in alpine ecosystems. Specifically, we first (i) applied a speciation‐based species delimitation framework to resolve taxonomic uncertainties, assign lineages to species, and delineate their respective distributions. Second, (ii) we tested for historical events of genetic introgression and estimated divergence times, providing critical insights into the pace of speciation and the extent to which extant lineages—likely recurrently brought into secondary contact during glacial expansions—have achieved partial or complete reproductive isolation. Then, (iii) we quantified population genetic structure and reconstructed demographic trajectories within each delimited species, integrating these inferences with expectations of past range dynamics derived from environmental niche modelling at fine temporal resolution. Finally, (iv) we tested the general hypothesis that microendemic species originate and accumulate more frequently at lower latitudes. We predict that populations at lower latitudes have experienced stronger demographic fluctuations and long‐term fragmentation during interglacials, thereby fuelling lineage diversification and microgeographic speciation. In contrast, the greater long‐term availability of suitable habitats and the more pronounced impacts of Pleistocene glaciations at higher latitudes likely enhanced connectivity and promoted more extensive range shifts, resulting in genetic homogenisation that ultimately constrained speciation and maintained genetic cohesiveness across broader distributional ranges (Jansson and Dynesius 2002).

## 2 Materials and Methods

### 2.1 Taxonomic and Population Sampling

During summers of 2021 and 2023, we sampled 34 populations that cover the entire distribution range of the genus *Oropodisma* Uvarov, 1942 (Cigliano et al. 2025; Table 1). Our sampling included most known populations from every described species and several populations with an uncertain taxonomic status (Willemse and Willemse 2008; Trillo and Ortego 2025). We aimed to collect five adult males and five adult females per population. However, the southernmost populations of the genus have experienced marked population declines in recent decades (Ortego 2025), and despite intensive prospecting during two sampling campaigns, we were only able to collect 2–5 individuals from some populations of *O. chelmosi* (MAEN, PARN) and *O. parnassica* (ELIK) (Table 1). Five specimens of *Podisma pedestris* (Linnaeus, 1758) were used as an outgroup in phylogenomic analyses. Whole specimens were preserved at −20°C in 1500 μL of 100% ethanol until needed for genomic and morphometric analyses. Further details on sampled taxa and populations are presented in Table 1.

**TABLE 1:** Geographical location of studied populations for the different species within the genus *Oropodisma*, including their taxonomic status before (putative) and after (assigned) species delimitation analyses (see Section 3.3).

Species (assigned)	Species (putative)	Population	Code	Latitude	Longitude	Elevation (m)	*n* (gen.)	*n* (morp.)
*O. macedonica*	*O. macedonica*	Mt. Ljuboten	(1) LJUB	42.19138	21.12681	1850	7	5
	*O. macedonica*	Popova Shapka^a^	(2) POPO	42.02277	20.87085	1990	8	5
	*O. macedonica*	Mavrovo National Park	(3) MAVR	41.79432	20.59325	2020	8	5
	*O. macedonica*	Galičica National Park	(4) GALI	40.94465	20.82640	1820	8	5
	*O. macedonica*	Mt. Valamara	(5) VALA	40.78335	20.49159	1850	8	5
	*O. macedonica*	Mt. Grammos	(6) GRAM	40.36023	20.80858	2030	7	5
	*O. macedonica*	Mt. Smolikas	(7) SMOL	40.08986	20.91012	2160	8	5
	*O. macedonica*	Mt. Vasilitsa	(8) VASI	40.04334	21.09396	1860	8	5
	*O. macedonica*	Mt. Mavrovouni	(9) MAVV	39.85602	21.15113	1960	8	5
	*O. macedonica*	Mt. Tymfi	(10) TYMF	39.97829	20.76881	1910	8	5
*O. tzoumerkae*	*O*. sp.	Mt. Kakarditsa	(11) KAKA	39.54484	21.18709	1860	8	5
	*O*. sp.	Mt. Tzoumerka^a^	(12) TZOU	39.40307	21.16325	1830	8	5
*O. karavica*	*O*. sp.	Mt. Avgo	(13) AVGO	39.48840	21.37764	1870	8	5
	*O. karavica*	Mt. Karava^a^	(14) KARA	39.32798	21.57219	1700	8	5
	*O. karavica*	Mt. Kazarma	(15) KAZA	39.30448	21.62246	1730	8	4
	*O*. sp.	Mt. Gavrogo	(16) GAVR	39.21393	21.28027	1730	8	5
*O. agrafae*	*O*. sp.	Mt. Agrafa^a^	(17) AGRA	39.14467	21.69589	1780	8	5
*O. lagrecai*	*O. lagrecai*	Mt. Triandafillia^a^	(18) TRIA	38.70310	21.68661	1770	8	5
*O. tymphrestosi*	*O. tymphrestosi*	Mt. Tymphristos^a^	(19) TYMP	38.93898	21.80771	1840	8	5
*O. willemsei*	*O*. sp.	Mt. Kaliakouda	(20) KALI	38.79402	21.76632	1740	8	5
	*O*. sp.	Mt. Oxia	(21) OXIA	38.78581	21.94144	1810	8	5
	*O. tymphrestosi*	Mt. Vardhousia	(22) VARD	38.68268	22.12990	2030	8	5
	*O. tymphrestosi*	Mt. Oiti	(23) OITI	38.81023	22.23648	1800	8	5
	*O. willemsei*	Mt. Giona–Koromilia^a^	(24) KORO	38.64453	22.32941	1790	8	1
	*O. willemsei*	Mt. Giona–Pirghakia^a^	(25) PIRG	38.59874	22.27364	2120	8	5
*O. parnassica*	*O. parnassica*	Mt. Parnassos^a^	(26) PARS	38.53077	22.61966	2280	8	1
	*O. parnassica*	Mt. Elikonas^a^	(27) ELIK	38.30000	22.88112	1710	4	5
*O. erymanthosi*	*O. erymanthosi*	Mt. Erymanthos^a^	(28) ERYM	37.95160	21.79381	1990	10	5
*O. kyllinii*	*O. kyllinii*	Mt. Kyllini^a^	(29) KYLL	37.93906	22.39612	2350	10	5
*O. chelmosi*	*O. chelmosi*	Mt. Panachaiko	(30) PANA	38.20706	21.86869	1730	10	5
	*O. chelmosi*	Mt. Chelmos^a^	(31) CHEL	37.97551	22.20285	2260	10	5
	*O. chelmosi*	Mt. Maenalon	(32) MAEN	37.64427	22.27626	1840	2	2
	*O. chelmosi*	Mt. Parnon	(33) PARN	37.27892	22.61227	1890	4	—
*O. taygetosi*	*O. taygetosi*	Mt. Taygetus^a^	(34) TAYG	36.95920	22.35185	2260	7	2

*Note:* Latitude and longitude are given in decimal degrees, and elevation is reported in meters above sea level.

Abbreviations: *n* (gen.), number of genotyped individuals; *n* (morp.), number of individuals used for geometric morphometric analyses.

^a^ Type localities.

### 2.2 Genomic Library Preparation and Genomic Data Processing

We extracted and purified DNA from each specimen using NucleoSpin Tissue kits (Macherey‐Nagel, Düren, Germany). We processed DNA into different genomic libraries using the double‐digestion restriction‐fragment‐based procedure (ddRAD‐seq) described in Peterson et al. (2012) and detailed in Methods S1. Raw sequences were demultiplexed and pre‐processed using stacks v. 2.66 (Rochette et al. 2019) and assembled using ipyrad v. 0.9.93 (Eaton and Overcast 2020). Methods S2 provides all details on sequence data filtering and assembling.

### 2.3 Phylogenomic Inference

First, we reconstructed the phylogenetic relationships among populations using three independent analytical approaches: the maximum‐likelihood (ML) method implemented in raxml v. 8.2.12 (Stamatakis 2014) and the coalescent‐based approaches implemented in svdquartets (Chifman and Kubatko 2014) and A01 analyses in bpp v. 4.7.0 (Flouri et al. 2018). Second, we used phylonetworks (Solís‐Lemus et al. 2017) to assess the potential presence and direction of post‐divergence gene flow, which might distort tree topology and/or result in unresolved phylogenetic relationships. Finally, the phylogenetic tree inferred using phylonetworks was fitted as the fixed topology in A00 analyses in bpp to estimate the posterior distribution of divergence times (τ; Flouri et al. 2018; Rannala and Yang 2003) among species defined according to species delimitation analyses in delineate (see Section 2.4). Five individuals of *P. pedestris* were used as an outgroup in raxml, svdquartets, and phylonetworks analyses. To reduce computational demands, we only considered a single representative individual per population—the one with the lowest proportion of missing data—to perform phylogenomic analyses. Methods S3 provides all details on the specific settings used to perform phylogenomic analyses.

### 2.4 Species Delimitation Analyses

We performed the constrained partitioned species delimitation analyses implemented in the programme delineate v. 1.2.3 (Sukumaran et al. 2021). Based on a population‐level ultrametric phylogeny and prior knowledge of species assignment for a subset of populations in the dataset, delineate estimates whether the remaining populations belong to an already described species or a new species (Sukumaran et al. 2021). We ran independent delineate analyses considering the slightly different topologies obtained using raxml, svdquartets, and bpp analyses (see Section 2.3 and Section 3.2). These topologies were fitted as fixed trees in independent A00 analyses in bpp to estimate the posterior distribution of divergence times and obtain ultrametric trees (Flouri et al. 2018). For details on analyses A00 in bpp, see Section 2.3 and Methods S3. For delineate analyses, we used as input these ultrametric trees and a species assignment reference file indicating whether each population belongs to a known nominal species (status = 1) or is assigned to an unknown species identity (status = 0). We made status assignments considering available taxonomic understanding of the system in conjunction with the evolutionary relationships between population lineages shown in the phylogeny (Sukumaran et al. 2021). Specifically, we assigned populations to a known or unknown nominal species status according to the literature and information on species distributions available at Orthoptera Species File (https://orthoptera.speciesfile.org/; Cigliano et al. 2025; see Table S1). Two populations originally assigned to the taxon *O. tymphrestosi* (OITI and VARD) were considered to have an unknown species identity, as they clustered in a clade including the type locality of *O. willemsei* (KORO and PIRG from Mt. Giona) and two other populations with an uncertain taxonomic status (KALI and OXIA) (see Table 1). We also assigned an unknown species identity to the two recently described species *O. tzoumerkae* and *O. agrafae* (Trillo and Ortego 2025).

### 2.5 Population Genetic Structure

We used the Bayesian Markov chain Monte Carlo clustering method implemented in the programme structure v. 2.3.3 (Pritchard et al. 2000) to quantify genetic structure and admixture across populations within each species identified in species delimitation analyses (see Section 3.3). These analyses were restricted to taxa including two or more genotyped populations (Table 1). Complementary to structure analyses, we performed principal component analyses (PCA) as implemented in the r v. 4.4.2 (R Core Team 2025) package ‘adegenet’ (Jombart 2008). We also estimated genetic differentiation between populations calculating the Weir & Cockerham weighted fixation index (*F*_ST_) as implemented in arlequin v. 3.5 (Excoffier and Lischer 2010). Methods S4 provides further details on the analyses of genetic structure.

### 2.6 Past Demographic History

We reconstructed the demographic history of each population using the programme stairway plot v. 2.1.2 (Liu and Fu 2020). Only populations with seven or more genotyped individuals were considered for these analyses (Table 1). To maximise the number of retained SNPs for the calculation of the SFS, we ran *step 7* from ipyrad separately for each specific population and retained loci that were represented in at least 50% of the individuals of the focal population (*min_samples_locus* = 50% of samples in the focal dataset). To remove all missing data for the calculation of the SFS and minimise errors in allele frequency estimates, each population was projected down to *n*‐2 diploids for each dataset of *n* genotyped individuals using the *easySFS.py* script (I. Overcast, https://github.com/isaacovercast/easySFS; accessed at 30/10/2024). We ran stairway plot considering one generation per year, assuming the mutation rate of 2.8 × 10^−9^ per site per generation estimated for *Drosophila melanogaster* (Keightley et al. 2014; see Methods S3 for details on the mutation rates employed), and performed 200 bootstrap replicates to estimate 95% confidence intervals.

### 2.7 Environmental Niche Modelling

We built environmental niche models (ENM) to reconstruct the geographic distribution of climatically suitable habitats for *Oropodisma* from the last glacial maximum (LGM; ca. 22 ka) to present. Specifically, we built an environmental niche model for the whole genus and species‐specific models for the three taxa— *O. macedonica* , *O. willemsei*, and *O. chelmosi*—for which a sufficient number of occurrence records were available for modelling their respective distributions. We used this information to infer distributional shifts experienced by the genus or each focal species in response to Quaternary climatic oscillations and determine across different time‐periods patterns of population fragmentation resulted from the spatial configuration of hypothetical barriers to dispersal (see Section 2.8). To build the ENM, we used the maximum entropy algorithm implemented in maxent v. 3.4.1 (Phillips et al. 2006; Phillips and Dudik 2008), available occurrence records, and the 19 bioclimatic layers (30‐arcsec resolution) from the CHELSA database (http://chelsa‐climate.org/bioclim/; Karger et al. 2017). To estimate environmental suitability from the LGM to present, we projected ENMs to bioclimatic conditions during the last 22,000 years at 100‐year time intervals (i.e., from 1990 ce to the LGM, for a total of 220 snapshots) using bioclimatic layers available at a high resolution (30‐arcsec) from the CHELSA‐TraCE21k v. 1.0 database (https://chelsa‐climate.org/; Karger et al. 2023). Further details on ENM are presented in Methods S5.

### 2.8 Landscape Genetic Analyses

We applied a landscape genetic approach to analyse a comprehensive set of factors that could explain genetic differentiation (*F*_ST_) among populations of *O. macedonica* , the only taxon with a wide distribution and a sufficient number of populations to perform robust statistical analyses (*n* = 10; Table 1). Pairwise *F*_ST_ values were calculated as detailed in Section 2.5. We tested the following explanatory variables: (i) geographical distance (GD); (ii) weighted topographic distance (WTD; Wang 2020); and (iii) isolation‐by‐resistance distances (IBR; McRae 2006; McRae and Beier 2007) based on a ‘flat landscape’ (IBR_FLAT_; i.e., all cells have equal resistance value = 1) and the configuration of environmentally suitable areas as inferred from projections of the ENM (see Section 2.7) to present‐day bioclimatic conditions (IBR_CURRENT_) and a layer of climatic suitability stability from the LGM to present (IBR_STABILITY_). We used multiple matrix regressions with randomisation (MMRR; Wang 2013) to test the matrix of population genetic differentiation (*F*_ST_) against GD, WTD and IBR matrices. Further details on landscape genetic analyses are presented in Methods S6.

### 2.9 Phenotypic Evolution Analyses

We used a geometric morphometric approach to quantify differences among populations and species in the shape of the phallus apex and furculae of males, two traits of taxonomic value in *Oropodisma* and likely involved in reproductive isolation (see Trillo and Ortego 2025 and references therein). See Methods S7 for further details on geometric morphometric analyses. Then, we used the backbone tree obtained with phylonetworks to estimate the phylogenetic signal in the first two principal components summarising landmarks and semilandmarks coordinates for each trait (phallus apex: PC1p and PC2p; furculae: PC1f and PC2f; see Methods S7), using Pagel's lambda (*λ*), as measured with the *phylosig* function of the ‘phytools’ package (Revell 2024) in r. Pagel's *λ* quantifies the magnitude of phylogenetic signal of a continuous trait assumed to be evolving under Brownian motion (Pagel 1999), where 0 indicates data with no phylogenetic signal (random noise), and 1 indicates strong fit to the Brownian Motion model. Next, we projected the phylogeny onto the shape space of the principal components to obtain a phylomorphospace (Sidlauskas 2008). Lastly, we determined the absolute fit of a Brownian Motion (BM) model to our morphological data as well as alternative models of trait evolution that allowed for evolutionary constraints (Ornstein‐Uhlenbeck [OU] model of bounded evolution), rate variability through time (“delta” model), and different degree of phylogenetic signal (“lambda” model) (Hernández et al. 2013). The delta model examines whether species traits evolution has accelerate or slowed down over time (*δ* = 1 indicate gradual evolution; *δ* < 1 indicate temporally early trait evolution or ‘early burst’, indicative of adaptive radiation; *δ* > 1 indicate temporally latter trait evolution, indicative of species‐specific adaptation), whereas the lambda model evaluates the extent to which the phylogeny correctly predicts patterns of trait similarity (*λ* = 1 indicate that phylogenetic relationship predict effectively the patterns of similarity between species traits; *λ* = 0 indicate that patterns of trait similarity among species are independent of phylogeny). Evolutionary models were fitted using the r package ‘geiger’ (Pennell et al. 2014). Models were compared based on their corrected Akaike information criteria (AICc) weights.

### 2.10 Determinants of Geographic Range Size

We first examined whether demographic instability and the extent of genetic differentiation among conspecific populations are associated with the latitudinal distribution of species. Then, we tested how these three variables explain differences in contemporary range size across taxa. The latitudinal distribution of each species was calculated as the geographic centroid of latitude values across its respective populations. Global genetic differentiation (*F*_ST_) among conspecific populations was estimated using the r package ‘hierfstat’. Demographic instability was calculated as the coefficient of variation of effective population size (*N*_e_) estimated over time for each population using stairway plot (see Section 2.6). When more than one population was available per species, values were averaged across all conspecific populations.

Contemporary range sizes were estimated from the ENM built for the genus *Oropodisma* (see Section 2.7), by counting the number of grid cells classified as suitable habitat within the known distribution range of each species. Suitability was defined as areas where the probability of *Oropodisma* occurrence exceeded the maximum training sensitivity plus specificity (MTSS) logistic threshold in maxent (Liu et al. 2005; for details, see Methods S5).

To assess the effects of explanatory variables on response variables while accounting for phylogenetic non‐independence, we used univariate phylogenetic generalised least squares (PGLS) models implemented in the r package ‘ape’ (with ‘nlme’) using the *gls* function. We performed PGLS analyses under a Brownian motion model of trait evolution (*λ* = 1) to avoid errors in estimating *λ* from a small phylogeny, especially for models involving global genetic differentiation, which can only be estimated for species represented by at least two populations (*n* = 6; Table 1). Analyses based on genetic summary statistics (i.e., gene differentiation and demographic stability) included a variance weighting term inversely proportional to the number of populations analysed for each species.

## 3 Results

### 3.1 Genomic Datasets

After the different quality filtering steps, the average number of reads retained per individual was 3,528,844 (range = 529,081–5,761,027). Attributes of the different genomic datasets used for all downstream analyses are presented in Table S2. All datasets have been deposited in Figshare (https://doi.org/10.6084/m9.figshare.29233733).

### 3.2 Phylogenomic Inference

**FIGURE 1:**
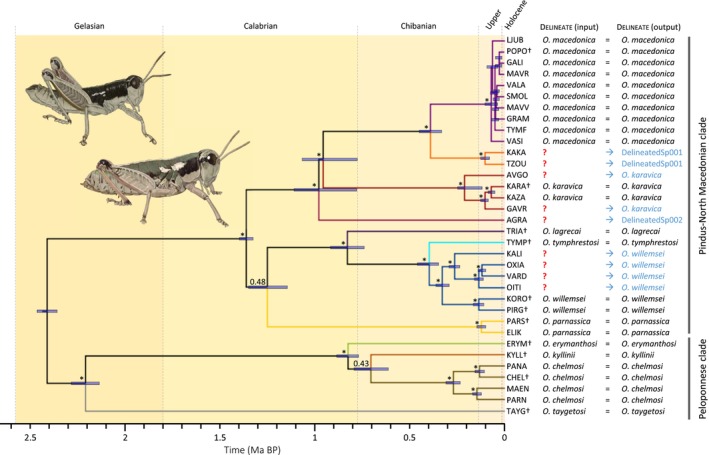
Phylogenetic tree inferred with bpp (analysis A01) and divergence times estimated using bpp (analysis A00) for the analysed populations of *Oropodisma*. Bayesian posterior probabilities estimated with bpp are indicated on the nodes (* > 0.95). Node support for the separation of *O. macedonica* populations is generally low and not shown on the tree. Bars on nodes indicate 95% highest posterior densities (HPD) of divergence times estimated considering a genomic mutation rate of 2.8 × 10^−9^ per site per generation and a one‐year generation time. Background colours indicate geological divisions of the Quaternary. The taxonomic status of populations before (? = unknown) and after species delimitation analyses in delineate is indicated. Population codes as described in Table 1, with crosses denoting type localities. Inset images show a male (top) and female (bottom) of *O. chelmosi* (illustrations by Marina Trillo).

**FIGURE 2:**
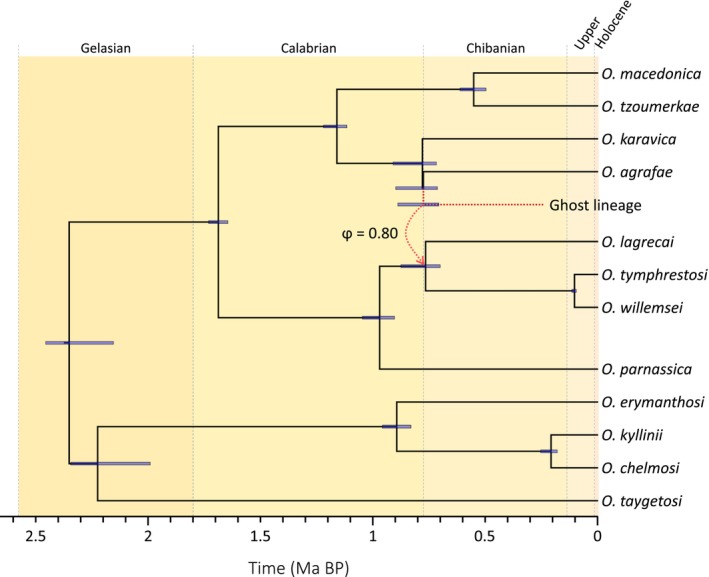
Species tree inferred with phylonetworks and divergence times and introgression probability (ϕ) estimated using bpp (analysis A00). Bars on nodes indicate 95% highest posterior densities (HPD) of divergence times estimated considering a genomic mutation rate of 2.8 × 10^−9^ per site per generation and a one‐year generation time. Background colours indicate geological divisions of the Quaternary.

Phylogenetic reconstructions with raxml, svdquartets and bpp yielded similar topological relationships, with only small differences involving some recently diverged populations within *O. macedonica* and *O. willemsei* (Figure 1; Figures S1 and S2). Phylogenetic trees showed the presence of two main clades, one including the taxa from the Peloponnese Peninsula (hereafter, the Peloponnese clade) and another including the taxa distributed north of the Corinthian Gulf (hereafter, the Pindus‐North Macedonian clade). The Peloponnese clade included a highly divergent lineage corresponding to the southernmost distributed taxon *O. taygetosi*, which was sister to a subclade including the species *O. erymanthosi*, *O. chelmosi*, and *O. kyllinii*. The second main clade encompassed two sister subclades, one including the taxa from the southern Pindus range in Central Greece (*O. lagrecae*, *O. tymphrestosi*, *O. willemsei*, and *O. parnassica*) and another including the taxa distributed across the northern Pindus range in Greece, Valamara massif in Albania, and Galičica and Shar Mountains in North Macedonia ( *O. macedonica* , *O. tzoumerkae*, *O. karavica*, and *O. agrafae*). Some populations originally assigned to *O. tymphrestosi* (OITI, VARD) were placed in a subclade that was sister to the type population (KORO and PIRG from Mt. Giona) of *O. willemsei*. All phylogenetic analyses supported the monophyly of the rest of species. Some populations with an uncertain taxonomic status clustered together with *O. tzoumerkae* (KAKA), *O. karavica* (AVGO, GAVR), and *O. willemsei* (KALI, OXIA). Node support was generally lower in svdquartets than in raxml and bpp analyses (Figure 1; Figures S1 and S2). Most internal nodes showed high support in both raxml and bpp analyses, except the node separating *O. parnassica* from its sister subclade including *O. lagrecae*, *O. tymphrestosi*, and *O. willemsei* in both raxml and bpp analyses, and the node separating *O. chelmosi* from *O. kyllinii* in bpp analyses (Figure 1; Figures S1 and S2). Nodes separating the recently diverged populations of *O. macedonica* were poorly supported (Figure 1; Figures S1 and S2).

phylonetworks analyses based on species delimited by delineate (see Section 3.3) revealed that models involving reticulation events (*h* > 0) fit our data better than models considering a strictly bifurcating tree (*h* = 0) (Figure S3). Negative pseudo‐likelihood scores decreased sharply from *h* = 0 to *h* = 1, with small improvements from *h* = 1 to *h* = 5, indicating that the best‐fitting phylogenetic model includes one introgression event (Figure S3). Phylogenetic relationships among taxa inferred by phylonetworks were similar to those obtained with raxml, svdquartets and bpp. The only exception was *O. agrafae*, which was inferred to be sister to the subclade including *O. karavica*, *O. macedonica* , and *O. tzoumerkae* in raxml, svdquartets, and bpp analyses but placed as a sister species of *O. karavica* by phylonetworks (Figure 2). phylonetworks analyses for *h* = 1 identified an introgression event (γ) from a ghost lineage sister to *O. agrafae* to the ancestor of *O. lagrecae*, *O. tymphrestosi*, and *O. willemsei* (Figure 2), with ~31% of gene copies in the ancestor of this subclade traced to the ghost lineage (Figure 2).

Estimation of divergence times in bpp (analysis A00), based on the most supported phylogenetic network, revealed that the Peloponnese clade and the Pindos‐North Macedonian clade diverged at the onset of the Pleistocene, approximately 2.35 million years ago (Ma; 95% highest posterior density [HPD]: 2.18–2.44 Ma) (Figure 2). The highly divergent taxon *O. taygetosi* split from the remaining species within the Peloponnese clade around 2.22 Ma (95% HPD: 2.00–2.32 Ma), whereas the divergence between the two main subclades within the Pindos–North Macedonian clade occurred at 1.62 Ma (95% HPD: 1.56–1.67 Ma) (Figure 2). The introgression event (ϕ) from a ghost lineage sister to *O. agrafae* into the ancestor of *O. lagrecai*, *O. tymphrestosi*, and *O. willemsei* was estimated to have taken place around 0.79 Ma (95% HPD: 0.73–0.90 Ma), with approximately 80% of the gene copies in the ancestor of this subclade traced to the ghost lineage (Figure 2). The divergence of the remaining lineages and subclades occurred between 1.16 and 0.11 Ma, with the most recent event corresponding to the split between *O. tymphrestosi* and *O. willemsei* (Figure 2).

### 3.3 Species Delimitation Analyses

Species delimitation analyses in delineate identified the recently described taxa *O. tzoumerkae* and *O. agrafae* as new species and assigned the rest of the taxonomically uncertain populations to the different delineated species: KAKA was assigned to *O. tzoumerkae*, AVGO and GAVR to *O. karavica*, and KALI, OXIA, VARD and OITI to *O. willemsei* (Table 1; Figure 1; Figures S1 and S2). Inferences from species delimitation were consistent considering the slightly different topological relationships among populations obtained using raxml, svdquartets and bpp (Figure 1; Figures S1 and S2).

### 3.4 Population Genetic Structure

**FIGURE 3:**
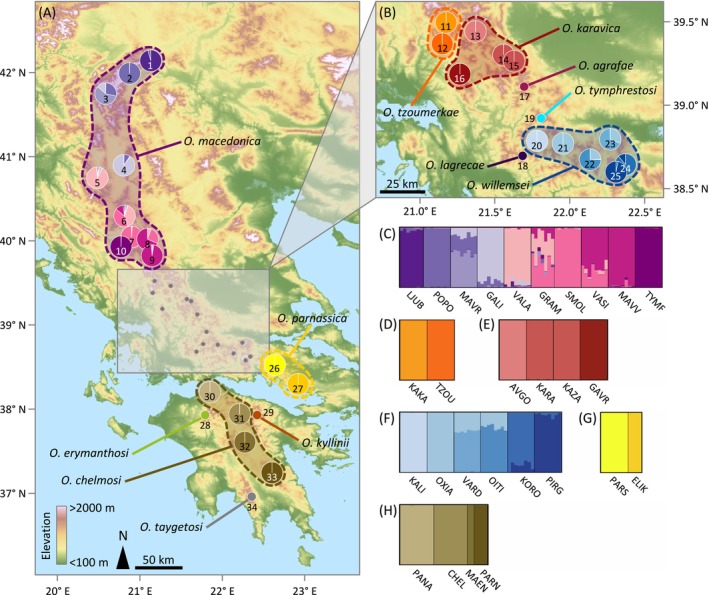
Geographic location of studied populations for each taxon within the genus *Oropodisma* and results of genetic assignments based on the Bayesian clustering method implemented in the programme structure. (A, B) Pie charts on maps and (C–H) barplots show the genetic assignments for populations of (C) *O. macedonica* , (D) *O. tzoumerkae*, (E) *O. karavica*, (F) *O. willemsei*, (G) *O. parnassica*, and (H) *O. chelmosi*. In barplots, each individual is represented by a vertical bar partitioned into *K* coloured segments showing the individual's probability of belonging to the cluster with that colour; thin vertical black lines separate individuals from different populations. Results of structure for each taxon are shown for the value of *K* at which the log probability of the data (LnPr(X|*K*)) reached a plateau (see Figure S4). Results for other *K* values are presented in Figures S5–S10. Population codes as described in Table 1. Maps display a digital elevation model (DEM) at 90‐m resolution derived from NASA's Shuttle Radar Topography Mission (SRTM) (https://portal.opentopography.org/).

structure analyses revealed pronounced genetic structure in all multi‐population species (Figure 3; Figures S4–S10), with most populations forming distinct genetic clusters and only a few cases of admixture or shared ancestry involving nearby populations of *O. karavica*, *O. willemsei*, and especially the more widely distributed *O. macedonica* (Figures S5–S10). Despite small sample sizes in three populations (PARN and MAEN in *O. chelmosi*, and ELIK in *O. parnassica*; Table 1), structure recovered them as distinct clusters, indicating robust genetic structure, though results should be interpreted with caution. Further details of the results for structure analyses are provided in Results S1. Principal component analyses (PCA) of genetic variation showed a genetic clustering of populations similar to that inferred by structure analyses at the different hierarchical levels for each species (Figures S5–S10). Estimates of genetic differentiation (*F*_ST_) between populations of each species are presented in Table S3. Except the nearby populations VASI and GRAM from *O. macedonica* , all other pairwise *F*_ST_ values were statistically significantly different from zero (Table S3). Pairwise *F*_ST_ values ranged between 0.041 for the nearby populations VASI‐MAVV of *O. macedonica* and 0.738 for the populations PANA‐PARN of *O. chelmosi* (Table S3).

### 3.5 Past Demographic History

**FIGURE 4:**
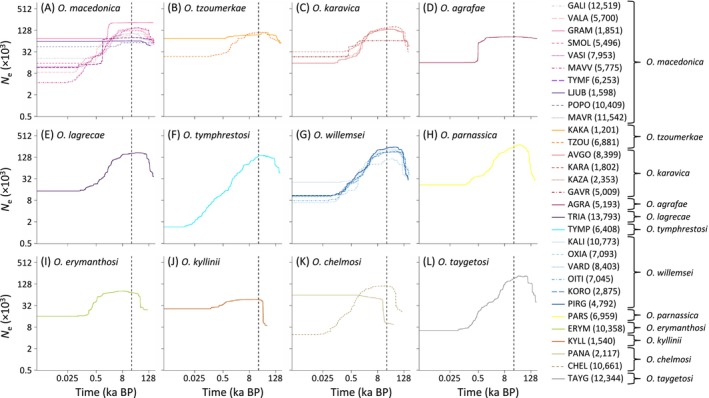
Demographic history of the studied populations for each taxon within the genus *Oropodisma* inferred using stairway plot. Only populations with *n* ≥ 7 genotyped individuals were analysed. Panels show the median of effective population size (*N*_e_) through time, estimated assuming a mutation rate of 2.8 × 10^−9^ and one generation per year (both axes on a logarithmic scale). Vertical dashed line indicates the Last Glacial Maximum (LGM; ~21,000 years ago). The number of polymorphic SNPs used to calculate the site frequency spectrum (SFS) for each population is indicated in parentheses. Population codes as described in Table 1.

stairway plot analyses showed that most populations of *Oropodisma* have experienced parallel demographic trajectories, undergoing severe declines of *N*_e_ generally starting at the onset of the Holocene preceded, in most cases, by demographic expansions during the last glacial period (Figure 4). The only exceptions are populations KAKA from *O. tzoumerkae*, KYLL from *O. kyllinii*, and PANA from *O. chelmosi*, which have experienced demographic stasis through time (KAKA) or expansions around the last glacial maximum (LGM) followed by demographic stability until the present day (KYLL and PANA) (Figure 4).

### 3.6 Environmental Niche Modelling

**FIGURE 5:**
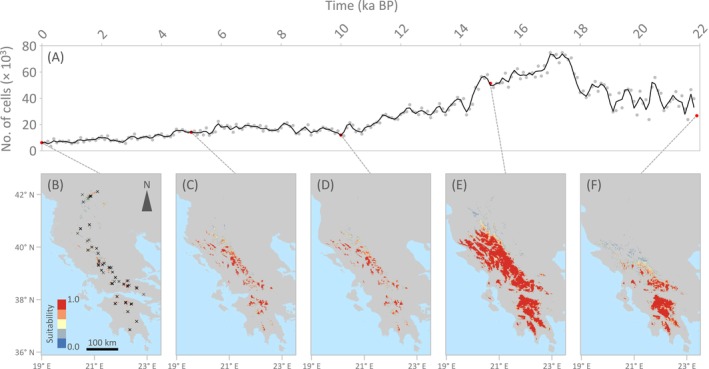
Extent of climatically suitable habitats for the genus *Oropodisma* as inferred from projections of the environmental niche model (ENM) to bioclimatic conditions during the last 22,000 years (i.e., from 1990 ce to the last glacial maximum, LGM) at 100‐year time intervals. (A) The availability of suitable habitats at each time interval was calculated as the number of cells where the probability of presence of *Oropodisma* is higher than the maximum training sensitivity plus specificity (MTSS) logistic threshold. (B–F) Maps show the distribution of climatically suitable habitats for the genus at five temporal snapshots (red dots in panel A), including (B) the present (0 ka; crosses show occurrence points used for ENM), (D) Holocene Climate Optimum (ca. 10 ka) and (F) LGM (ca. 22 ka).

Climatically suitable areas predicted by the ENM are highly congruent with the present‐day observed distribution of the genus *Oropodisma*, which currently forms small and severely fragmented populations on mountain tops (Figure 5b). Projections of the ENM onto bioclimatic conditions from the LGM to the present at 100‐year intervals revealed that the extent and connectivity of suitable areas, as identified using the maximum training sensitivity plus specificity threshold for species presence (Liu et al. 2005), peaked at the end of the last glacial period (ca. 17 ka) and gradually declined from then on (Figure 5). Our reconstructions since the LGM showed that distributional shifts were mostly limited to elevational displacements, alternating periods of population fragmentation and connectivity at local/regional scales, but with the presence of at least some refugial populations persisting in all mountain ranges where *Oropodisma* is distributed today (Figure 5). Note that only tiny areas—barely visible in Figure 5c,f—were predicted to be suitable across most time periods in the northernmost portion of the contemporary distribution of the genus (i.e., present‐day populations of the taxon *O. macedonica* ), suggesting the long‐term persistence of populations in small local refugia or frequent extinction‐recolonisation dynamics. Remarkably, suitable habitats from the Peloponnese Peninsula have remained continuously disconnected from the rest of the Balkans at least since the LGM, supporting its long‐term isolation (Figure 5). Temporal dynamics in the extent and connectivity of climatically suitable habitats inferred for species‐specific models of *O. macedonica* (Figure S11), *O. willemsei* (Figure S12), and *O. chelmosi* (Figure S13) yielded qualitatively similar results than the genus‐based predictions. For details on the performance and parameters of ENMs, see Table S4.

### 3.7 Landscape Genetic Analyses

**FIGURE 6:**
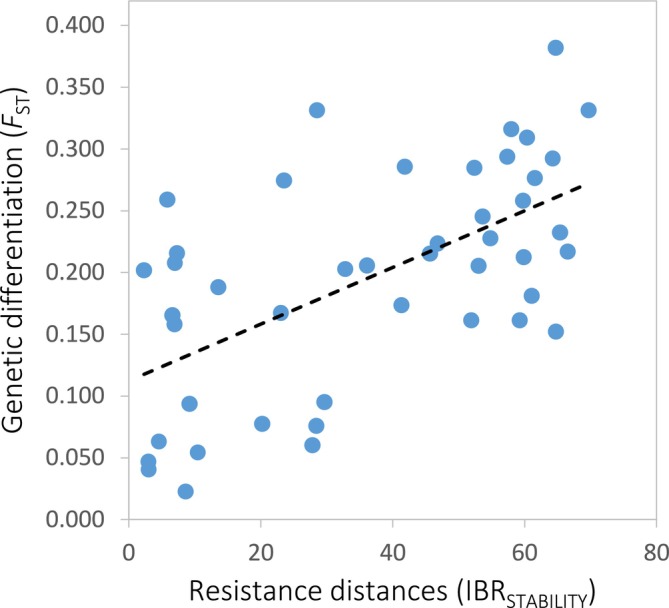
Relationship between genetic differentiation (*F*_ST_) between populations of *Oropodisma macedonica* and resistance distances defined by habitat suitability stability from the last glacial maximum (LGM) to present (IBR_STABILITY_). Regression line is shown.

Univariate matrix regressions with randomisation showed that genetic differentiation between populations of *O. macedonica* is significantly correlated with geographical distance (GD), weighted topographic distances (WTD), and resistance distances defined by a flat landscape (IBR_FLAT_) and habitat suitability stability from the LGM to present (IBR_STABILITY_) (all *p*‐values < 0.01; Table S5), but not with resistance distances defined by the contemporary distribution of environmentally suitable habitats (IBR_CURRENT_; *p* = 0.192; Table S5). However, IBR_STABILITY_ was the best fit to our data (*R* ^2^ = 0.353) and the only predictor retained in the final multivariate model (Table 2; Figure 6).

**TABLE 2:** Multiple matrix regression with randomisation for genetic differentiation (*F*_ST_) between populations of *Oropodisma macedonica* in relation to (i) geographical distance, (ii) weighted topographic distance, and resistance distances defined by (iii) a flat landscape (IBR_FLAT_; i.e., all cells have equal resistance value = 1), (iv) contemporary habitat suitability (IBR_CURRENT_), and (v) habitat suitability stability from the last glacial maximum (LGM) to present (IBR_STABILITY_).

Variable	*β*	*t*	*p*
Explanatory terms			
Constant	0.109		
IBR_STABILITY_	0.545	4.84	< 0.001
Rejected terms			
Geographical distance		0.47	0.402
Weighted topographic distance		−0.67	0.659
IBR_FLAT_		0.27	0.441
IBR_CURRENT_		−5.33	0.998

Abbreviations: *p*, significance level; *t*, *t*‐statistic; *β*, regression coefficient.

### 3.8 Geometric Morphometric Analyses

Population pairwise Euclidean distances between least‐squares means for shape variation of the phallus apex (Table S6; Figure S14a) and the furculae (Table S7; Figure S14b) of males revealed non‐significant differences among conspecific populations, indicating morphological cohesiveness within taxa. Species pairwise Euclidean distances for genitalia (Table S8) and the furculae (Table S9) of males showed that most taxa were significantly differentiated in one or both traits. Aside from comparisons involving *O. taygetosi*—which were limited by small sample size (*n* = 2; Table 1)—the only non‐significant differences were observed among certain taxa within the Pindos–North Macedonian clade (*O. tzoumerkae*‐*O. karavica*‐*O. lagrecai*, *O. karavica*‐*O. tymphrestosi*, *O. tymphrestosi*‐*O. willemsei*, and *O. tymphrestosi*‐*O. parnassica*) and within the Peloponnese clade (*O. erymanthosi*‐*O. kyllinii*) (Tables S8 and S9).

### 3.9 Phenotypic Evolution Analyses

All phenotypic traits showed a moderately low and non‐significant phylogenetic signal (PC1p: λ ~0, *p* = 1; PC2p: λ = 0.61, *p* = 0.06; PC1f: λ = 0.57, *p* = 0.11; PC2f: λ = 0.28, *p* = 0.66) (Figures S15 and S16). The phymorphospace for genitalia shape showed that *O. agrafae* occupies a distinctive position in relation to the remaining lineages, whereas *O. kyllinii*, *O. erymanthosi*, and *O. chelmosi* tend to cluster together in both genitalia and furculae morphospaces (Figures S15 and S16). For PC1p and PC1f, the trait evolution model receiving the strongest support was the lambda model (Table 3), although for PC1f, the Brownian motion (BM) model was equally favoured. The unequivocal support for the lambda model with λ≈0 in PC1p indicates phylogenetic independence (i.e., no concordance between phylogeny and trait values; Table 3). A single‐rate BM model, in which traits evolve via a random‐walk process at a constant rate (*σ* ^2^), provided the best fit for PC2p and PC2f, though in the former, the lambda model was equally supported (Table 3). In the case of PC2f, the low phylogenetic signal (*λ*≈0.28) suggests that phylogenetic relationships may influence trait evolution, but not in a manner strictly proportional to divergence time, as assumed by BM. Nonetheless, BM was preferred due to its lower complexity.

**TABLE 3:** Relative support for alternative evolutionary models for shape variation of the phallus apex (PC1p, PC2p) and the furculae (PC1f and PC2f) of males in *Oropodisma*.

Trait	Model	AIC_c_	∆ AIC_c_	wtAIC_c_	σ ^2^	*delta*	*lambda*
PC1p	BM	19.20	6.08	0.04	27.60		
	OU	22.87	9.75	0.01			
	*Delta*	17.84	4.72	0.08	11.44	3.99	
	*Lambda*	13.12	0	0.86	27.60		0
PC2p	BM	−6.30	0	0.36	3.29		
	OU	−2.76	3.54	0.06			
	*Delta*	−5.20	1.10	0.21	1.67	2.99	
	*Lambda*	−6.29	0.01	0.36	3.29		0.61
PC1f	BM	−19.69	0.01	0.37	0.47		
	OU	−16.14	3.56	0.06			
	*Delta*	−18.53	1.17	0.20	0.55	2.99	
	*Lambda*	−19.70	0	0.37	0.47		0.57
PC2f	BM	−29.65	0	0.49	1.07		
	OU	−26.07	3.58	0.08			
	*Delta*	−28.28	1.37	0.25	0.24	2.99	
	*Lambda*	−27.68	1.97	0.18	1.07		0.28

Abbreviations: AIC_c_, Corrected Akaike Information Criterion; BM, Pure Brownian motion; Delta (*δ*), gradual vs. non‐gradual evolution; Delta, Pagel's delta; Lambda (*λ*), phylogenetic signal; Lambda, Pagel's lambda; OU, Ornstein‐Uhlenbeck model; wtAIC_c_, Akaike weights; σ ^2^, evolutionary rate.

### 3.10 Determinants of Geographic Range Size

**FIGURE 7:**
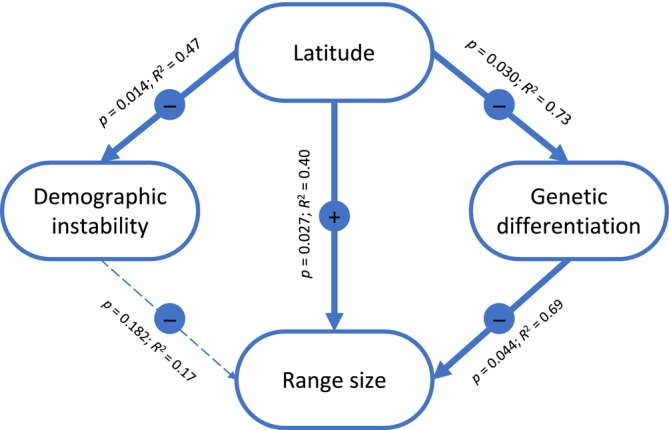
Schematic representation of phylogenetic generalised least squares (PGLS) analyses testing the correlations between latitude, demographic instability, genetic differentiation, and range size across species of the genus *Oropodisma*. Arrows link explanatory (top) to response (bottom) variables and circles indicate the sign of the correlation. Analyses assume a Brownian motion model (*λ* = 1) and those based on genetic summary statistics (i.e., genetic differentiation and demographic instability) incorporate a variance weighting term inversely proportional to the sample size available for each species (i.e., number of genotyped populations; Table 1). Analyses of genetic differentiation are based on the subset of species with two or more sampled populations (Table 1). *p* = significance level; *R* ^2^ = coefficient of determination.

Both genetic differentiation (*β* = −0.106 ± 0.032, *t* = −3.29, *p* = 0.030) and demographic instability (*β* = −9.00 ± 3.01, *t* = −2.99, *p* = 0.014) were negatively associated with latitude (Figure 7). In turn, range size was positively associated with latitude (*β* = 582.86 ± 224.97, *t* = 2.59, *p* = 0.027) and negatively associated with genetic differentiation (*β* = −4818.33 ± 1620.72, *t* = −2.97, *p* = 0.041), whereas no significant association was found with demographic instability (*β* = −23.86 ± 16.63, *t* = −1.43, *p* = 0.182) (Figure 7).

## 4 Discussion

Alpine ecosystems provide unique natural laboratories to study the processes that promote the formation of new species in a context of shifting distributions and ephemeral geographical isolation. In this scenario, secondary contact and recurrent gene flow are expected to hinder the completion of speciation, whereas small population sizes and habitat instability jeopardise the long‐term persistence of newly formed lineages (Rahbek et al. 2019). High rates of lineage formation triggered by geographical isolation (i.e., frequent splitting), the presence of climate refugia that prevent extinction (i.e., local persistence), and the rapid evolution of reproductive isolation that impedes incipiently diverging lineages from merging (i.e., short speciation times) are likely key processes driving the extraordinary levels of local microendemism that characterise alpine and montane biotas at mid to low latitudes (Dynesius and Jansson 2014; e.g., Tzedakis 2009; Feliner 2014). Our study, which integrates phylogenomic‐based species delimitation analyses, inference of demographic dynamics, and spatial modelling, provides fundamental insights into the rates at which new lineages arise and how they persist through evolutionary time until the formation of full‐fledged species. Multiple lines of evidence suggest that the balance between rates of lineage formation and the extent of gene flow resulted from the interplay between habitat connectivity‐fragmentation and shifting distributions driven by Pleistocene climatic oscillations is critical to explaining rates of species formation and why most of them accumulate at lower latitudes.

### 4.1 Species Delimitation and Range Size Delineation

Species delimitation analyses identified twelve evolutionarily independent lineages, including populations with previously uncertain taxonomic status that were assigned either to the ten traditionally recognised species (Cigliano et al. 2025) or to two recently described taxa (Trillo and Ortego 2025) (Table 1; Figure 1; Figures S1 and S2). Although populations assigned to the same taxon by delineate were phenotypically cohesive, several closely related species pairs within major clades did not exhibit clear diagnostic differences in the traits examined. This highlights the inherent challenges of taxonomic delimitation in recent radiations, where cryptic or quasi‐cryptic species are common and morphological divergence may lag behind lineage divergence (e.g., Huang et al. 2020; Ortego et al. 2024). The resulting taxonomic assignments enabled a refined delineation of species distributions and revealed pronounced heterogeneity in range size among taxa, with the northernmost distributed species occupying an area nearly as large as that of all other congeneric species combined (Figure 3). In contrast, most species exhibited extreme microendemism, with very small areas of occupancy (< 100 km^2^) restricted to one or a few nearby mountaintops. These results illustrate the value of speciation‐based species delimitation approaches for resolving taxonomic uncertainties and accurately mapping species distributions (Sukumaran et al. 2021). By improving taxonomic resolution, such approaches help prevent the misidentification of populations from obscuring conservation priorities and biasing downstream ecological and evolutionary inferences (Hey et al. 2003; Isaac et al. 2004; Bickford et al. 2007).

### 4.2 A Prime Geographic Dimension of Speciation

Phylogenomic reconstructions revealed a predominant geographic dimension of speciation, with two major clades estimated to have diverged around the onset of the Pleistocene (ca. 2.6 Ma; Figures 1 and 2). These clades correspond to taxa distributed north of the Corinthian Gulf and within the Peloponnese Peninsula (Figure 2). This pattern is consistent with allopatric divergence documented in multiple organismal groups across the Corinthian Gulf (see Kiourtsoglou et al. 2021, and references therein), a long‐standing biogeographic barrier associated with the isolation of the Peloponnese Peninsula from the rest of the Balkans since the Pliocene (Collier and Dart 1991). Within the Peloponnese clade, a deep split (> 2 Ma) isolates a narrowly endemic lineage restricted to Mount Taygetos, highlighting this mountain system as a significant centre of phylogenetic endemism (see also Pires et al. 2024). Phylogenetic network analyses further revealed a reticulation event, with a ghost lineage contributing to the ancestry of a subclade distributed in the Pindus Range. Such a ghost lineage may represent either an extinct species or an unsampled taxon yet to be discovered (Ottenburghs 2020; Tricou et al. 2022). Although sampling was extensive and encompassed nearly all known populations, environmental niche modelling indicates that additional suitable but unsurveyed areas remain, particularly in isolated high‐elevation massifs of the central Pindus range. Given the pronounced microendemism characteristic of this and other alpine radiations, these regions represent promising targets for future surveys and the potential discovery of previously unrecognised evolutionary diversity within the focal complex.

### 4.3 Drivers of Range Size and Formation of Microendemic Species

Inferred patterns of genetic differentiation, demographic reconstructions, and spatially explicit landscape genetic analyses provide valuable insights into the proximate processes driving microgeographic diversification in alpine organisms from temperate regions (e.g., Ortego and Knowles 2022; Ortego et al. 2024). Environmental niche modelling indicates that taxa experienced severe range contractions during interglacial periods, leading to population fragmentation and marked reductions in effective population size (*N*_e_) (Figure 4). Consistent with this, populations show strong genetic structure (Figure 3), a pattern widely reported in alpine organisms with limited dispersal ability (e.g., Slatyer et al. 2014; Ortego et al. 2021; Martínez‐Sañudo et al. 2022; Ortego and Knowles 2022). Such genetic fragmentation is expected to contribute to high rates of lineage formation (i.e., frequent splitting; Dynesius and Jansson 2014), triggering allopatric speciation during periods of isolation in interglacial refugia (Bennett and Provan 2008; e.g., DeChaine and Martin 2005; Ortego and Knowles 2022). Despite this pervasive fragmentation, demographic trajectories and levels of genetic differentiation vary markedly among taxa (Figure 3). Both genetic differentiation and the magnitude of demographic fluctuations decrease with latitude relative to the geographic range of the focal complex, suggesting greater opportunities for lineage formation at lower latitudes, where populations tend to be more isolated and experience stronger temporal fluctuations in *N*_e_ (Figure 7). Given nearly identical life‐history traits and ecological requirements across taxa, reduced genetic differentiation at higher relative latitudes is best explained by greater long‐term habitat availability and connectivity, combined with more extensive range shifts driven by Pleistocene climatic oscillations. Larger and more connected habitat networks, together with higher climatic instability at more northern latitudes within the range of the complex, are expected to enhance gene flow and homogenise gene pools at timescales shorter than those required for reproductive barriers to evolve (see Section 3.4). This, in turn, may reduce the likelihood of microgeographic speciation and favour the persistence of wider distributional ranges (Jansson and Dynesius 2002; Morueta‐Holme et al. 2013). Accordingly, range size is negatively associated with genetic differentiation and positively correlated with latitude, providing a mechanistic explanation for the higher incidence of local microendemism toward the southern limits of the complex distribution (Figure 7). These findings illustrate how the interplay between climatic history, geographic context, and habitat connectivity can generate contrasting outcomes in genetic structure, demographic stability, and diversification across latitudinal gradients in alpine systems from temperate regions.

### 4.4 Rapid Evolution of Reproductive Isolation

In systems characterised by recurrent range shifts and episodes of secondary contact, the rapid evolution of complete or partial reproductive isolation is likely critical for the persistence of recently formed lineages and the completion of speciation. Divergence times inferred here indicate that some species originated within the last few hundred thousand years (Figure 2), a timescale comparable to that reported for other insect taxa that are demonstrably reproductively isolated (Coyne and Orr 1989; Coyne and Orr 2004; Ortego et al. 2021, 2024). Despite adjacent distributions and likely secondary contact during glacial range expansions (Figure 5), phylogenetic network analyses detected very limited evidence of historical hybridisation, supporting the existence of effective reproductive barriers among taxa. Given the broad similarity in ecological requirements across taxa, reproductive isolation during early stages of divergence is most plausibly driven by genetic drift and the accumulation of mutations in small, geographically isolated populations during interglacial periods. These processes are expected to facilitate the evolution of both prezygotic (e.g., divergence in mating signals or behaviours; Panhuis et al. 2001; Ritchie 2007) and postzygotic barriers (e.g., Dobzhansky‐Muller genetic incompatibilities or chromosomal rearrangements; Orr 1995; Rieseberg 2001; Presgraves 2010), with secondary contact potentially reinforcing isolation when hybrid fitness is reduced (Servedio and Noor 2003; Pfennig 2016). The lack of consistent differentiation in genital morphology among closely related taxa suggests that these traits have not acted as primary drivers of reproductive isolation. Instead, speciation may have been promoted by the rapid accumulation of cryptic genetic incompatibilities (e.g., Barton 1980; Hagberg et al. 2022; Castillo et al. 2017; Boman et al. 2025). The generally low phylogenetic signal in the traits examined further indicates that their evolution is shaped by a combination of stochastic and context‐dependent selective processes, rather than being strictly constrained by shared ancestry.

## 5 Conclusions

Our results indicate that range shifts and increased connectivity among populations at comparatively northern latitudes cause incipient lineages formed during periods of isolation to merge at rates that exceed the time required for speciation to complete (Jansson and Dynesius 2002; e.g., Maier et al. 2019). These processes maintain the genetic and phenotypic cohesion across broad geographic areas, thereby constraining opportunities for microgeographic speciation at higher latitudes. Consequently, the formation of microendemic versus wide‐ranging species appears to be governed by an unstable equilibrium between lineage splitting and fusion, shaped by the heterogeneous latitudinal impacts of Pleistocene climatic oscillations on population demographic and range dynamics (Jansson and Dynesius 2002). Our findings support the notion that this context‐dependent balance determines whether glacial–interglacial cycles promote or interrupt speciation along latitudinal gradients in temperate mountain regions. Collectively, this framework helps reconcile the long‐standing debate on the evolutionary consequences of Quaternary climatic fluctuations and their potential role as either drivers or inhibitors of speciation (Klicka and Zink 1997; Dynesius and Jansson 2000; Jansson and Dynesius 2002; e.g., Maier et al. 2019; Ortego and Knowles 2022; Marques et al. 2024).

## Author Contributions

J.O. conceived the study. J.O., M.T., V.G.‐N. designed the research. J.O. and J.G.‐R. collected the samples. M.T. prepared the genomic libraries and obtained phenotypic data. J.O., M.T. and V.G.‐N. analysed the data. J.O. led the writing with inputs from all the authors.

## Funding

This work was supported by Ministerio de Ciencia, Innovación y Universidades, PID2021‐123298NB‐I00, TED2021‐129328B‐I00, PID2025‐168840NB‐I00. European Social Fund, PID2021‐123298NB‐I00, PID2025‐168840NB‐I00. NextGenerationEU, TED2021‐129328B‐I00.

## Conflicts of Interest

The authors declare no conflicts of interest.

## Supporting information


**Data S1:** mec70332‐sup‐0001‐Supinfo01.docx.
**Table S1:** Taxonomic status of populations considered for delineate analyses.
**Table S2:** Attributes of genomic datasets obtained for each studied species.
**Table S3:** Genetic differentiation between populations.
**Table S4:** Summary of environmental niche modelling.
**Table S5:** Univariate matrix regressions with randomisation for *Oropodisma macedonica*.
**Table S6:** Population pairwise Euclidean distances for shape variation of the phallus apex of males.
**Table S7:** Population pairwise Euclidean distances for shape variation of the furculae of males.
**Table S8:** Species pairwise Euclidean distances for shape variation of the phallus apex of males.
**Table S9:** Species pairwise Euclidean distances for shape variation of the furculae of males.
**Figure S1:** Phylogenetic tree inferred with raxml and divergence times estimated using bpp.
**Figure S2:** Phylogenetic tree inferred with svdquartets and divergence times estimated using bpp.
**Figure S3:** Summary of model fit with phylonetworks.
**Figure S4:** Log probability of the data and the magnitude of Δ*K* for structure analyses.
**Figure S5:** Principal component analysis (PCAs) of genetic variation and genetic assignments based on structure analyses for *Oropodisma macedonica*.
**Figure S6:** Principal component analysis (PCAs) of genetic variation and genetic assignments based on structure analyses for *Oropodisma tzoumerkae*.
**Figure S7:** Principal component analysis (PCAs) of genetic variation and genetic assignments based on structure analyses for *Oropodisma karavica*.
**Figure S8:** Principal component analysis (PCAs) of genetic variation and genetic assignments based on structure analyses for *Oropodisma willemsei*.
**Figure S9:** Principal component analysis (PCAs) of genetic variation and genetic assignments based on structure analyses for *Oropodisma parnassica*.
**Figure S10:** Principal component analysis (PCAs) of genetic variation and genetic assignments based on structure analyses for *Oropodisma chelmosi*.
**Figure S11:** Environmental niche modelling for *Oropodisma macedonica*.
**Figure S12:** Environmental niche modelling for *Oropodisma willemsei*.
**Figure S13:** Environmental niche modelling for *Oropodisma chelmosi*.
**Figure S14:** Principal component analyses (PCA) for shape variation of the phallus apex and the furculae of males.
**Figure S15:** Phylomorphospace plot for shape variation of the phallus apex and the furculae of males.
**Figure S16:** Reconstructed evolution of shape variation of the phallus apex and the furculae of males.
**Methods S1**. Genomic library preparation.
**Methods S2**. Genomic data filtering and assembling.
**Methods S3**. Phylogenomic analyses.
**Methods S4**. Population genetic structure.
**Methods S5**. Environmental niche modelling.
**Methods S6**. Landscape genetic analyses.
**Methods S7**. Geometric morphometric analyses.
**Results S1**. structure analyses.

## Data Availability

Raw Illumina reads have been deposited at the NCBI Sequence Read Archive (SRA) under BioProject PRJNA1390705. Input files for all analyses are available for download on Figshare (https://doi.org/10.6084/m9.figshare.29233733).
