# Supplementary material for: Latitudinal Clines in Gene Flow and Demographic Stability Reveal Drivers of Microendemism in a Radiation of Alpine Grasshoppers
Source: Mol Ecol. 2026 Apr 3;35(7):e70332. doi: 10.1111/mec.70332 (PMC13047727; doi:10.1111/mec.70332)
Supplement: Supplementary file 1 — Data S1: mec70332‐sup‐0001‐Supinfo01.docx. Table S1: Taxonomic status of populations considered for delineate analyses. Table S2: Attributes of genomic datasets obtained for each studied species. Table S3: Genetic differentiation between populations. Table S4: Summary of environmental niche modelling. Table S5: Univariate matrix regressions with randomisation for Oropodisma macedonica. Table S6: Population pairwise Euclidean distances for shape variation of the phallus apex of males. Table S7: Population pairwise Euclidean distances for shape variation of the furculae of males. Table S8: Species pairwise Euclidean distances for shape variation of the phallus apex of males. Table S9: Species pairwise Euclidean distances for shape variation of the furculae of males. Figure S1: Phylogenetic tree inferred with raxml and divergence times estimated using bpp. Figure S2: Phylogenetic tree inferred with svdquartets and divergence times estimated using bpp. Figure S3: Summary of model fit with phylonetworks. Figure S4: Log probability of the data and the magnitude of ΔK for structure analyses. Figure S5: Principal component analysis (PCAs) of genetic variation and genetic assignments based on structure analyses for Oropodisma macedonica. Figure S6: Principal component analysis (PCAs) of genetic variation and genetic assignments based on structure analyses for Oropodisma tzoumerkae. Figure S7: Principal component analysis (PCAs) of genetic variation and genetic assignments based on structure analyses for Oropodisma karavica. Figure S8: Principal component analysis (PCAs) of genetic variation and genetic assignments based on structure analyses for Oropodisma willemsei. Figure S9: Principal component analysis (PCAs) of genetic variation and genetic assignments based on structure analyses for Oropodisma parnassica. Figure S10: Principal component analysis (PCAs) of genetic variation and genetic assignments based on structure analyses for Oropodisma chelmosi. Figure S11: Environm [file MEC-35-e70332-s001.docx]

Supporting Information for

**Latitudinal clines in gene flow and demographic stability reveal drivers of microendemism in a radiation of alpine grasshoppers**

Joaquín Ortego, Marina Trillo, Jorge Gutiérrez-Rodríguez, and Vicente García-Navas

Journal: *Molecular Ecology*

**Contents:**

**Supplementary methods**

**METHODS S1** Genomic library preparation

**Methods S2** Genomic data filtering and assembling

**Methods S3** Phylogenomic analyses

**Methods S4** Population genetic structure

**Methods S5** Environmental niche modelling

**Methods S6** Landscape genetic analyses

**Methods S7** Geometric morphometric analyses

**Supplementary results**

**RESULTS S1** structure analyses

**Supplementary tables**

**Table S1** Taxonomic status of populations considered for delineate analyses

**TABLE S2** Attributes of genomic datasets obtained for each studied species

**Table S3** Genetic differentiation between populations

**table S4** Summary of environmental niche modelling

**table S5** Univariate matrix regressions with randomization for *Oropodisma macedonica*

**table S6** Population pairwise Euclidean distances for shape variation of the phallus apex of males

**table S7** Population pairwise Euclidean distances for shape variation of the furculae of males

**table S8** Species pairwise Euclidean distances for shape variation of the phallus apex of males

**table S9** Species pairwise Euclidean distances for shape variation of the furculae of males

**Supplementary figures**

**figure S1** Phylogenetic tree inferred with raxml and divergence times estimated using bpp

**figure S2** Phylogenetic tree inferred with svdquartets and divergence times estimated using bpp

**figure S3** Summary of model fit with phylonetworks

**figure S4** Log probability of the data and the magnitude of Δ*K* for structure analyses

**Figure S5** Principal component analysis (PCAs) of genetic variation and genetic assignments based on structure analyses for *Oropodisma macedonica*

**Figure S6** Principal component analysis (PCAs) of genetic variation and genetic assignments based on structure analyses for *Oropodisma tzoumerkae*

**Figure S7** Principal component analysis (PCAs) of genetic variation and genetic assignments based on structure analyses for *Oropodisma karavica*

**Figure S8** Principal component analysis (PCAs) of genetic variation and genetic assignments based on structure analyses for *Oropodisma willemsei*

**Figure S9** Principal component analysis (PCAs) of genetic variation and genetic assignments based on structure analyses for *Oropodisma parnassica*

**Figure S10** Principal component analysis (PCAs) of genetic variation and genetic assignments based on structure analyses for *Oropodisma chelmosi*

**Figure S11** Environmental niche modelling for *Oropodisma macedonica*

**Figure S12** Environmental niche modelling for *Oropodisma willemsei*

**Figure S13** Environmental niche modelling for *Oropodisma chelmosi*

**Figure S14** Principal component analyses (PCA) for shape variation of the phallus apex and the furculae of males

**Figure S15** Phylomorphospace plot for shape variation of the phallus apex and the furculae of males

**Figure S16** Reconstructed evolution of shape variation of the phallus apex and the furculae of males

**Supplementary references**

**Supplementary methods**

**METHODS S1** Genomic library preparation

We processed genomic DNA into six genomic libraries (44-45 individuals/library) using the double-digestion restriction-fragment-based procedure (ddRAD-seq) described in Peterson et al. (2012). In brief, we digested DNA with the restriction enzymes MseI and EcoRI (New England Biolabs, Ipswich, MA, USA) and ligated Illumina adaptors including unique 7-bp barcodes to the digested fragments of each individual. We pooled ligation products, size-selected them between 475-580 bp with a Pippin Prep machine (Sage Science, Beverly, MA, USA), and amplified the fragments by PCR with 12 cycles using the iProofTM High-Fidelity DNA Polymerase (BIO-RAD, Veenendaal, The Netherlands). Paired-end 150-bp sequencing was performed on an Illumina NovaSeq6000 platform.

**METHODS S2** Genomic data filtering and assembling

We screened raw reads for quality with fastqc v. 0.11.5 (S. Andrews; <http://www.bioinformatics.babraham.ac.uk/projects/fastqc/>) and used trimmomatic v. 0.36 (Bolger et al., 2014) to remove low-quality base calls corresponding to enzyme cut sites in both R1 (138-bp after removing barcodes and EcoRI cut site) and R2 (146-bp after removing MseI cut site) Illumina reads. Raw sequences were demultiplexed and filtered for overall quality using the *process_radtags* program in stacks v. 2.66 (Rochette et al., 2019). We retained reads with a Phred score > 10 (using a sliding window of 15%), no adaptor contamination, and that had an unambiguous barcode. After quality filtering by *process_radtags*, we retained for downstream analyses those samples with >500k reads. We assembled our sequences into *de novo* loci using ipyrad v. 0.9.93 (Eaton & Overcast, 2020). Briefly, reads retained after *process_radtags* were further quality-filtered with ipyrad to convert base calls with a Phred score <20 into *N*s and discard reads with >5 *N*s (*max_low_qual_bases* = 5). Afterwards, we clustered the retained reads within- and across samples considering a threshold of sequence similarity of 85% (*clust_threshold* = 0.85) and discarded those clusters with a minimum coverage depth of less than 6 (*mindepth_majrule* = 6; *mindepth_statistical* = 6). Resulting loci shorter than 35 bp (*filter_min_trim_len* = 35), containing one or more heterozygous sites across more than 50% of individuals (*max_shared_Hs_locus* = 0.5) and showing more than 20% polymorphic sites (*max_SNPs_locus* = 0.2) were discarded. The choice of different filtering and assembling thresholds have a little impact on the obtained inferences (e.g., Eaton et al., 2015; González-Serna et al., 2019; Ortego et al., 2018). For this reason, unless otherwise indicated, all downstream analyses were performed using genomic datasets obtained with pyrad considering a clustering threshold of sequence similarity of 85 % (*clust_threshold* = 0.85) and discarding loci that were not present in at least 50 % individuals (*min_samples_locus* = 50 % of samples in each specific dataset).

**METHODS S3** Phylogenomic analyses

raxml – We ran raxml v. 8.2.12 (Stamatakis, 2014) using a matrix of concatenated SNPs (all SNPs per locus; *.snp* file from ipyrad) edited in the r v. 4.4.0 (R Core Team, 2025) package ‘phrynomics’ (B. Banbury, <http://github.com/bbanbury/phrynomics>). The input dataset contained 138,663 SNPs. We applied an ascertainment bias correction using the conditional likelihood method (Lewis, 2001), used a GTR-GAMMA model of nucleotide evolution, performed 100 rapid bootstrap replicates, and searched for the best-scoring maximum likelihood tree.

svdquartets – We ran svdquartets (Chifman & Kubatko, 2014) as implemented in paup* version 4.0a169 (Swofford, 2002). The input dataset contained 3,503 unlinked SNPs (*.usnp* file from ipyrad). We exhaustively evaluated all possible quartets and performed non-parametric bootstrapping with 100 replicates for quantifying topological uncertainty.

bpp (analysis A01) – We used whole ddRAD-seq loci (*.loci* file from ipyrad) to run tree estimation A01 analyses in bpp v. 4.7.0 (Flouri et al., 2018). The *.loci* file from pyrad was edited and converted into a bpp input file using a custom UNIX script (J. Ortego, <https://github.com/OrtegoLab/ipyrad2bpp>). As bpp analyses are computationally highly demanding, we limited the dataset to 1,000 loci. We set uniform rooted trees as species tree prior, applied an automatic adjustment of fine-tune parameters, set the diploid option to indicate that the input sequences are unphased, and adjusted the inverse-gamma distributions of *θ* (*α* = 3, *β* = 0.04) and *𝜏* (*α* = 3, *β* = 0.07) priors according to empirical estimates calculated based on the number of segregating sites per site (Huang et al., 2020). Note that bpp analyses do not require defining outgroup, as the program samples the root position along with the other nodes of the tree. We ran two independent replicate analyses for 400,000 generations, sampling every 2 generations, after a burn-in of 20,000 generations.

phylonetworks – We used Species Networks applying Quartets (snaq) implemented in phylonetworks (Solís-Lemus et al., 2017) to determine whether a strictly bifurcating tree (i.e., lack of post-divergence gene flow) or a phylogenetic network (i.e., one or more introgression events) better explains the divergence history of *Oropodisma*. snaq performs maximum pseudo-likelihood estimation of phylogenetic networks using the coalescent-based multispecies model and quartet-based concordance analyses (Solís-Lemus et al., 2017). This approach evaluates the most likely network, depicts the major phylogenetic topology (“major edge”) and past introgression events (“minor edges”), and calculates γ, the vector of inheritance probabilities describing the proportion of genes inherited by a hybrid node from one of its parents (Solís-Lemus et al., 2017). We first used magnet v. 0.1.5 pipeline (J.C. Bagley, <http://github.com/justincbagley/MAGNET>) to split each locus contained in the pyrad output file *.gphocs* into a separate phylip-formatted alignment file. We applied trimal v. 1.2 (Capella-Gutiérrez et al., 2009) to our phylip dataset in order to filter out loci with a high mean percentage of identity (>0.99) across the multisequence alignment and retain only those that are most informative (Bernardes et al., 2007). The final dataset contained 3,447 loci. Then, we inferred a maximum-likelihood (ML) gene tree for each locus using raxml with a GTR+GAMMA model and 100 bootstrap replicates and used these gene trees and phylonetworks to estimate quartet concordance factors (CFs), defined as the proportion of genes that support each possible relationship between each set of four taxa. We estimated the best phylogenetic network testing a varying number of reticulation events (*h* from 0 to 5), each optimized with 50 independent runs. To make phylonetworks analyses computationally tractable, we created a taxon map file in which each population from the same species was assigned to the same tip. Assignments of populations to species were defined according to species delimitation analyses detailed in section 2.4. The optimal number of reticulation events was chosen using a heuristic approach by plotting negative pseudo-likelihood scores against *h*-values, as recommended by Solís-Lemus et al. (2017).

bpp (analysis A00) – The phylogenetic tree inferred using phylonetworks was fitted as the fixed topology in A00 analyses in bpp to estimate the posterior distribution of divergence times (*τ*; Flouri et al., 2018; Rannala & Yang, 2003) among species defined according to species delimitation analyses in delineate (see section 2.4). We ran the analyses using the same dataset and settings considered for tree inference A01 analyses in bpp. In order to ensure the convergence of the runs (ESS > 200), we ran two independent replicate analyses for 2,000,000 generations, sampling every 2 generations, after a burn-in of 100,000 generations. We estimated divergence times using the equation 𝜏 = 2𝜇t, where 𝜏 is the divergence in substitutions per site estimated by bpp, 𝜇 is the per site mutation rate per generation, and t is the absolute divergence time in years (Walsh et al., 2001). We considered a mutation rate per site per generation of 2.8 × 10^-9^, estimated for *Drosophila melanogaster* based on whole-genome parent-offspring sequencing analyses (Keightley et al., 2014). This is similar to the spontaneous mutation rate inferred for the butterfly *Heliconius melpomene* using a comparable approach (2.9 × 10^-9^; Keightley et al., 2015) and is of the same order of magnitude (1.31 × 10^-9^ - 2.27 × 10^-9^) as indirect phylogenetic estimates of mutation rates obtained for Orthoptera based on transcriptomic data (Nabholz, 2024).

**Methods S4** Population genetic structure

We ran structure v. 2.3.3 (Pritchard et al., 2000) analyses with 200,000 MCMC cycles, following a burn-in step of 100,000 iterations, assuming correlated allele frequencies and admixture and without using prior population information. To reduce computational demands of structure analyses, we randomly selected a subset of a maximum of 10,000 SNPs for each species' dataset. We conducted 15 independent runs for each value of *K* genetic clusters, where *K* ranged from 1 to *n*+1 for each dataset of *n* populations, to estimate the most likely number of genetic clusters. We retained the 10 runs having the highest likelihood for each value of *K*. As recommended by Gilbert et al. (2012) and Janes et al. (2017), we used two statistics to interpret the number of genetic clusters (*K*) that best describes our data: log probabilities of the data (LnPr(X|*K*); Pritchard et al., 2000) and Δ*K* (Evanno et al., 2005), calculated as implemented in structure harvester (Earl & vonHoldt, 2012). We used clumpp v. 1.1.2 and the Greedy algorithm to align multiple runs of structure for the same *K* value (Jakobsson & Rosenberg, 2007) and distruct v. 1.1 (Rosenberg, 2004) to visualize the individuals’ probabilities of population membership in bar plots. Complementary to structure analyses, we performed principal component analyses (PCA) as implemented in the r v. 4.4.2 package ‘adegenet’ (Jombart, 2008). Before running PCAs, we replaced missing data by the mean allele frequency of the corresponding locus estimated across all samples (Jombart, 2008). We also estimated genetic differentiation between populations calculating the Weir & Cockerham weighted fixation index (*F*_ST_) as implemented in arlequin v. 3.5 (Excoffier & Lischer, 2010). We determined statistical significance with Fisher’s exact tests after 10,000 permutations, applying a false discovery rate (FDR) adjustment (5%, *q* < 0.05) to control for multiple tests.

**Methods S5** Environmental niche modelling

We built environmental niche models (ENM) using the maximum entropy algorithm implemented in maxent v. 3.4.1 (Phillips et al., 2006; Phillips & Dudik, 2008) and the 19 bioclimatic layers from the CHELSA database interpolated to 30-arcsec resolution for current conditions (<http://chelsa‐climate.org/bioclim/>; Karger et al., 2017). We used our own species occurrence data and records available in the literature and Orthoptera Species File (<http://orthoptera.speciesfile.org>; Cigliano et al., 2025). All occurrence records used for ENM are deposited in Figshare (<https://doi.org/10.6084/m9.figshare.29233733>). Prior to modelling, we mapped and examined all records to identify and exclude those representing obvious geo-referencing errors. After data filtering and excluding duplicates (i.e., records falling within the same grid cell), we retained 84 occurrence records for the whole genus *Oropodisma*, 24 records for *O. macedonica*, 10 records for *O. willemsei*, and 11 records for *O. chelmosi*. We used arcmap v. 10.8.2 (ESRI) to create a minimum convex polygon (MCP) around occurrence points. A 100-km buffer around this MCP was used as calibration background to sample pseudoabsences (*n* = 10,000 background points) in order to account for accessible areas that the focal taxon could potentially reach and colonize (Barve et al., 2011). We conducted parameter tuning and determined the optimal feature class (FC) and regularization multiplier (RM) settings for maxent using the r package ‘ENMeval’ (Muscarella et al., 2014) and the Akaike’s Information Criterion corrected for small sample size (AICc) (Warren & Seifert, 2011). We tested a total of 203 models of varying complexity by combining a range of regularization multipliers (RM) (from 1 to 15 in increments of 0.5) with seven different feature classes (FC) combinations (L, LQ, LQP, H, LQH, LQHP, LQHPT, where L = linear, Q = quadratic, H = hinge, P = product and T = threshold). We performed a three-stage approach to select model parameters (RM and FC) and the set of environmental variables retained in the final models (Warren et al., 2014; e.g., González-Serna et al., 2019). In a first step, we built a full set of models including all bioclimatic variables, retained the model with the lowest AICc score, and among those variables that were spatially correlated (Pearson’s coefficient > 0.9) we only retained for the next step the one with the highest percent contribution to the model. In a second step, we ran another full set of models with the subset of variables retained in the first step, selected the model with the lowest AICc score, and removed variables with zero percent contribution to the model. In a third step, we re-ran a final full set of models with the environmental variables retained in the previous steps and used for all downstream analyses the model with the lowest AICc score. Complementary, we evaluated the performance of the retained models using the “block” method for data partitioning into training and testing datasets (*n* = 500 iterations) (Muscarella et al., 2014). Specifically, we calculated the area under the receiver-operating characteristic plot on the testing data (AUC_TEST_) and the minimum training presence omission rate (OR_MTP_). An AUC_TEST_ value > 0.9 suggests a high discriminatory ability of the model (Peterson et al., 2011) whereas an OR_MTP_ close to zero is indicative of a low degree of model overfitting (Radosavljevic & Anderson, 2014).

**Methods S6** Landscape genetic analyses

We applied a landscape genetic approach to analyze a comprehensive set of factors that could explain genetic differentiation (*F*_ST_) among populations of *O. macedonica*. We tested the following explanatory variables:

(i) Geographical distance (GD): The geodesic distance between sampled populations, calculated using the r package ‘geodist’ (Padgham & Sumner, 2020).

(ii) Weighted topographic distance (WTD): We used the digital elevation model (DEM) at 90 meters resolution from the NASA´s Shuttle Radar Topographic Mission (SRTM) (<https://portal.opentopography.org/>) to calculate weighted topographic distances between each pair of populations, as implemented in the r package ‘topodistance’ (Wang, 2020). We calculated weighted topographic paths using the *topoWeightedDist* function, with a linear function to weight angle of aspect changes and an exponential function to weight the slope between cells, as recommended by Wang (2020). These topographic distances account for the additional overland distance covered by an organism due to elevation changes imposed by topographic relief and assume that the energetic cost to traverse a slope varies exponentially with the change in angle.

(iii) Isolation-by-resistance (IBR): We built spatially-explicit IBR scenarios of population connectivity based on the configuration of environmentally suitable areas as inferred from projections of the ENM to present-day bioclimatic conditions (IBR_CURRENT_) and a layer of climatic suitability stability (IBR_STABILITY_). Climatic suitability stability was calculated as the proportion of time-intervals that a given pixel was predicted to be suitable for the focal species from the LGM to present. To this end, we projected the species-specific ENM at 100-year time intervals as detailed in section 2.8 and used the r package ‘raster’ to transform suitability maps for each time period to binary layers (presence = 1; absence = 0) using the maximum training sensitivity plus specificity (MTSS) logistic threshold of maxent (Liu et al., 2005). Resistance distances between all pairs of populations were calculated under each scenario using an eight-neighbor cell connection scheme in circuitscape v. 4.0.5 (McRae, 2006, McRae & Beier, 2007). As resistance distances cannot be calculated between totally isolated populations (i.e., conductance = 0), we transformed zero pixel values to very small conductance values (=0.0001) before running circuitscape. We also calculated resistance distances based on a ‘flat landscape’ (IBR_FLAT_; i.e., all cells have equal resistance value = 1), which is analogous to geographical distance but more appropriate for comparison with others competing models also generated with circuitscape (e.g., Noguerales et al., 2016).

We used multiple matrix regressions with randomization (MMRR; Wang 2013) to test the matrix of population genetic differentiation (*F*_ST_) against GD, WTD and IBR matrices. Given that geographical, topographic and resistance distances are only expected to have a positive effect on the degree of genetic differentiation between populations, we used one-tailed hypothesis tests for making statistical decisions regarding the null hypothesis of no effect of independent variables on genetic differentiation (Ruxton & Neuhäuser, 2010; e.g., Yannic et al., 2018). The model was initially constructed with all explanatory terms fitted (i.e., a full model) and final model was selected using a backward-stepwise procedure by progressively removing non-significant variables (starting with the least significant ones) until all retained terms within the model were significant. Then, we tested the significance of the rejected terms against this model to ensure that no additional variable reached significance. The result is the minimal most adequate model for explaining the variability in the response variable, where only the significant explanatory terms are retained (e.g., Ortego et al., 2015).

**Methods S7** Geometric morphometric analyses

We extracted the internal male genitalia, took images of the phallus apex and furculae, and digitized landmarks and semilandmarks as detailed in Trillo & Ortego (2025). We used the Generalized Procrustes Analysis (GPA; Rohlf, 1998) implemented in the r package ‘geomorph’ (Adams & Otarola‐Castillo, 2013) to remove the effects of scale, rotation, and translation of all landmark and semilandmark configurations. Then, we performed a principal component analysis (PCA) on the covariance matrix of Procrustes-aligned data to identify the major axes of shape variation among individuals and summarize patterns of morphological variation for the two traits. We retained the first two principal components (PC), which explained 74.52 % of the variance for phallus apex (PC1p: 58.03 %; PC2p: 16.49 %) and 62.95% of the variance for furculae (PC1f: 40.73%; PC2f: 22.22 %). We used the function *ProcD.lm* in the r package ‘geomorph’ to perform a Procrustes ANOVA (Anderson, 2001) and assess differences in shape variation among populations and species (Adams & Otarola‐Castillo, 2013). Finally, we used the function *pairwise* in the r package ‘rrpp’ to calculate the distance and corresponding empirically derived *p*-values between each pair of populations and species using a nonparametric randomized residual permutation procedure (Collyer & Adams, 2018).

**Supplementary results**

**RESULTS S1** structure analyses

structure analyses for *O. macedonica* identified the most likely number of genetic clusters as *K* = 2 according to the Δ*K* criterion, but LnPr(X|*K*) sharply increased from *K* = 1 to *K* = 4 and steadily from *K* = 4 to *K* = 8 (Figure S4a). For *K* = 2, the two genetic clusters tended to separate populations from Mt. Shar (POPO, LJUB), Mt. Korab (MAVR) and Mt. Galičica (GALI) in North Macedonia from the rest of the populations, with a spatial cline of shared ancestry linked to the geographical proximity of populations (Figure S5b). From *K* = 3 to *K* = 8, populations split hierarchically in different genetic clusters with variable degrees of shared ancestry involving geographically close populations (Figure S5b). In *O. tzoumerkae*, LnPr(X|*K*) increased from *K* = 1 to *K* = 2 and sharply declined from *K* = 2 to *K* = 3 (Figure S4b), showing the presence of two genetic clusters that separate the two populations of the species with no signatures of genetic admixture (Figure S6b). structure analyses for *O. karavica* identified the most likely number of genetic clusters as *K* = 2 according to the Δ*K* criterion, but LnPr(X|*K*) reached a plateau at *K* = 3 (Figure S4c). Populations from Mt. Avgo (AVGO), Mt. Gavrogo (GAVR) and northern Agrafa range (KARA and KAZA) split hierarchically into different genetic clusters from *K* = 2 to *K* = 3, with no signatures of genetic admixture (Figure S7b). The two nearby populations from northern Agrafa range (KARA and KAZA) —separated by <5km— were assigned to the same genetic cluster at both *K* = 3 and *K* = 4 (Figure S7b). structure analyses for *O. willemsei* identified the most likely number of genetic clusters as *K* = 2 according to the Δ*K* criterion, but LnPr(X|*K*) sharply increased from *K* = 1 to *K* = 3 and steadily from *K* = 3 to *K* = 6 (Figure S4d). Analyses for *K* = 2 separated the easternmost populations from Mt. Giona (KORO and PIRG) from the rest of the populations, with different degrees of admixed ancestry in populations (OITI, VARD) located in contact zones between the two genetic clusters (Figure S8b). structure analyses for *O. parnassica* showed that LnPr(X|*K*) sharply increased from *K* = 1 to *K* = 2 and reached a plateau at *K* = 2 (Figure S4e), revealing the presence of two genetic clusters that fully separated the two populations of the species with no signatures of genetic admixture (Figure S9b). Finally, structure analyses for *O. chelmosi* identified the most likely number of genetic clusters as *K* = 3 according to the Δ*K* criterion, but LnPr(X|*K*) sharply increased until *K* = 4 (Figure S4f). The species presented a hierarchical latitudinal genetic structure, and for *K* = 4 every population was assigned to a different cluster with no signatures of genetic admixture (Figure S10b).

**Supplementary tables**

**TABLE S1** Studied populations for the different species within the genus *Oropodisma*, including their putative taxonomic status according to the literature and taxonomic status assignment (1 = known; 0 = unknown) considered for species delimitation analyses in delineate. Crosses denote type localities.

| Population | Code | Putative species | Status | References |
| --- | --- | --- | --- | --- |
| Mt. Ljuboten | (1) LJUB | *O. macedonica* | 1 | 1 |
| Popova Shapka | (2) POPO† | *O. macedonica* | 1 | 1-5 |
| Mavrovo National Park | (3) MAVR | *O. macedonica* | 1 | 1 |
| Galičica National Park | (4) GALI | *O. macedonica* | 1 | 1 |
| Mt. Valamara | (5) VALA | *O. macedonica* | 1 | 1 |
| Mt. Grammos | (6) GRAM | *O. macedonica* | 1 | 6 |
| Mt. Smolikas | (7) SMOL | *O. macedonica* | 1 | 7-8 |
| Mt. Vasilitsa | (8) VASI | *O. macedonica* | 1 | 9 |
| Mt. Mavrovouni | (9) MAVV | *O. macedonica* | 1 | 8, 10 |
| Mt. Tymfi | (10) TYMF | *O. macedonica* | 1 | 8, 10-11 |
| Mt. Kakarditsa | (11) KAKA | *O.* sp. | 0 | 9 |
| Mt. Tzoumerka | (12) TZOU† | *O. tzoumerkae* | 0 | 9 |
| Mt. Avgo | (13) AVGO | *O.* sp. | 0 | 9 |
| Mt. Karava | (14) KARA† | *O. karavica* | 1 | 8-9, 12 |
| Mt. Kazarma | (15) KAZA | *O. karavica* | 1 | 9 |
| Mt. Gavrogo | (16) GAVR | *O.* sp.*.* | 0 | 9 |
| Mt. Agrafa | (17) AGRA† | *O. agrafae* | 0 | 9 |
| Mt. Triandafillia | (18) TRIA† | *O. lagrecai* | 1 | 7-9 |
| Mt. Tymphristos | (19) TYMP† | *O. tymphrestosi* | 1 | 1-2, 8, 10 |
| Mt. Kaliakouda | (20) KALI | *O.* sp. | 0 | 9 |
| Mt. Oxia | (21) OXIA | *O.* sp. | 0 | 9 |
| Mt. Vardhousia | (22) VARD | *O. tymphrestosi* | 0 | 1, 7-8 |
| Mt. Oiti | (23) OITI | *O. tymphrestosi* | 0 | 7-8 |
| Mt. Giona - Koromilia | (24) KORO† | *O. willemsei* | 1 | 8-9, 12 |
| Mt. Giona - Pirghakia | (25) PIRG† | *O. willemsei* | 1 | 8-9, 12 |
| Mt. Parnassos | (26) PARS† | *O. parnassica* | 1 | 1, 4-5, 8, 10, 13 |
| Mt. Elikonas | (27) ELIK | *O. parnassica* | 1 | 9 |
| Mt. Erymanthos | (28) ERYM† | *O. erymanthosi* | 1 | 1-2, 5, 8 |
| Mt. Kyllini | (29) KYLL† | *O. kyllinii* | 1 | 1-2, 4-5, 8 |
| Mt. Panachaiko | (30) PANA | *O. chelmosi* | 1 | 8, 12 |
| Mt. Chelmos | (31) CHEL† | *O. chelmosi* | 1 | 1, 4-5, 8 |
| Mt. Maenalon | (32) MAEN | *O. chelmosi* | 1 | 7-8 |
| Mt. Parnon | (33) PARN | *O. chelmosi* | 1 | 7-8 |
| Mt. Taygetus | (34) TAYG† | *O. taygetosi* | 1 | 1, 8, 10 |

References – 1: Cigliano et al., 2025; 2: Machácková & Fikácek, 2014; 3: Ramme, 1951; 4: Uvarov, 1942; 5: Willemse, 1971; 6: Lemonnier-Darcemont et al., 2015; 7: Willemse, 1979; 8: Willemse, 1984; 9: Willemse & Willemse, 2008; 10: Willemse, 1972a; 11: Willemse, 1972b; 12: La Greca & Messina, 1977; 13: Scudder, 1987

**TABLE S2** Attributes of genomic datasets used for different analyses, including number of individuals in the ingroup/outgroup and number of retained reads and loci after different filtering steps in stacks and ipyrad.

|  |  |  |  | Number of retained reads | |  |  |
| --- | --- | --- | --- | --- | --- | --- | --- |
| Dataset | Ingroup | Outgroup | Usage | Mean | Range |  | Loci |
| Phylogenomic | 34 | 5 | raxml, svdquartets, phylonetworks | 4,033,053 | 1,204,245-5,425,722 |  | 3,506 |
| Phylogenomic | 34 | – | bpp | 4,033,053 | 1,204,245-5,425,722 |  | 5,860 |
| *O. macedonica* | 78 | – | structure, PCA, arlequin | 3,607,702 | 662,241-5,547,267 |  | 15,311 |
| *O. tzoumerkae* | 16 | – | structure, PCA, arlequin | 2,560,422 | 529,081-5,172,011 |  | 7,212 |
| *O. karavica* | 32 | – | structure, PCA, arlequin | 3,486,736 | 874,774-5,761,027 |  | 10,651 |
| *O. willemsei* | 48 | – | structure, PCA, arlequin | 3,727,663 | 2,166,212-5,185,509 |  | 8,398 |
| *O. parnassica* | 12 | – | structure, PCA, arlequin | 3,243,318 | 2,263,852-5,503,714 |  | 19,517 |
| *O. chelmosi* | 26 | – | structure, PCA, arlequin | 3,530,829 | 1,698,242-5,445,621 |  | 10,736 |

**TABLE S3** Population pairwise *F*_ST_ values (below the diagonal) and their corresponding *q*-values (above the diagonal) for populations (*n* ≥ 4) of (A) *O. macedonica*, (B) *O. tzoumerkae*, (C) *O. karavica*, (D) *O. willemsei*, (E) *O. parnassica*, and (F) *O. chelmosi*. Significance of pairwise *F*_ST_ values was determined with Fisher´s exact tests after 10,000 permutations, as implemented in arlequin v. 3.5. Statistically significant *F*_ST_ values after false discovery rate adjustment (FDR) to control for multiple tests (FDR of 5%, *q* < 0.05) are indicated in bold. Population codes as described in Table 1.

| (A) *O. macedonica* | | | | | | | | | | |
| --- | --- | --- | --- | --- | --- | --- | --- | --- | --- | --- |
| Code | LJUB | POPO | MAVR | GALI | VALA | GRAM | SMOL | VASI | MAVV | TYMF |
| LJUB | – | 0.000 | 0.000 | 0.000 | 0.000 | 0.001 | 0.001 | 0.000 | 0.000 | 0.000 |
| POPO | **0.259** | – | 0.000 | 0.000 | 0.000 | 0.000 | 0.000 | 0.000 | 0.000 | 0.000 |
| MAVR | **0.216** | **0.202** | – | 0.000 | 0.001 | 0.000 | 0.000 | 0.000 | 0.000 | 0.001 |
| GALI | **0.332** | **0.275** | **0.167** | – | 0.000 | 0.000 | 0.000 | 0.000 | 0.000 | 0.000 |
| VALA | **0.224** | **0.286** | **0.174** | **0.206** | – | 0.001 | 0.000 | 0.000 | 0.000 | 0.000 |
| GRAM | **0.294** | **0.285** | **0.161** | **0.215** | **0.078** | – | 0.001 | 0.152 | 0.005 | 0.000 |
| SMOL | **0.233** | **0.309** | **0.213** | **0.245** | **0.076** | **0.094** | – | 0.001 | 0.001 | 0.000 |
| VASI | **0.152** | **0.258** | **0.161** | **0.205** | **0.060** | 0.023 | **0.047** | – | 0.001 | 0.000 |
| MAVV | **0.217** | **0.277** | **0.181** | **0.228** | **0.095** | **0.054** | **0.063** | **0.041** | – | 0.000 |
| TYMF | **0.332** | **0.382** | **0.292** | **0.316** | **0.203** | **0.188** | **0.208** | **0.166** | **0.158** | – |
|  |  |  |  |  |  |  |  |  |  |  |
| (B) *O. tzoumerkae* | | |  |  |  |  | | | | |
| Code | KAKA | TZOU |  |  |  |  |  |  |  |  |
| KAKA | – | 0.000 |  |  |  |  |  |  |  |  |
| TZOU | **0.328** | – |  |  |  |  |  |  |  |  |
|  |  |  |  |  |  |  |  |  |  |  |
| (C) *O. karavica* | | | | |  |  |  |  |  |  |
| Code | AVGO | KARA | KAZA | GAVR |  |  |  |  |  |  |
| AVGO | – | 0.001 | 0.000 | 0.000 |  |  |  |  |  |  |
| KARA | **0.583** | – | 0.000 | 0.000 |  |  |  |  |  |  |
| KAZA | **0.614** | **0.132** | – | 0.000 |  |  |  |  |  |  |
| GAVR | **0.651** | **0.345** | **0.381** | – |  |  |  |  |  |  |
|  |  |  |  |  |  |  |  |  |  |  |
| (D) *O. willemsei* | | | | | | |  |  | | |
| Code | KALI | OXIA | VARD | OITI | KORO | PIRG |  |  |  |  |
| KALI | – | 0.000 | 0.001 | 0.000 | 0.000 | 0.000 |  |  |  |  |
| OXIA | **0.717** | – | 0.000 | 0.000 | 0.000 | 0.000 |  |  |  |  |
| VARD | **0.609** | **0.351** | – | 0.000 | 0.000 | 0.000 |  |  |  |  |
| OITI | **0.590** | **0.317** | **0.193** | – | 0.000 | 0.000 |  |  |  |  |
| KORO | **0.667** | **0.692** | **0.602** | **0.535** | – | 0.000 |  |  |  |  |
| PIRG | **0.659** | **0.679** | **0.597** | **0.550** | **0.212** | – |  |  |  |  |
|  |  |  |  |  |  |  |  |  |  |  |
| (E) *O. parnassica* | | |  |  |  |  |  |  |  |  |
| Code | PARS | ELIK |  |  |  |  |  |  |  |  |
| PARS | – | 0.002 |  |  |  |  |  |  |  |  |
| ELIK | **0.329** | – |  |  |  |  |  |  |  |  |
|  |  |  |  |  |  |  |  |  |  |  |
| (F) *O. chelmosi* | | | |  |  |  |  |  |  |  |
| Code | PANA | CHEL | PARN |  |  |  |  |  |  |  |
| PANA | – | 0.000 | 0.001 |  |  |  |  |  |  |  |
| CHEL | **0.330** | – | 0.001 |  |  |  |  |  |  |  |
| PARN | **0.738** | **0.438** | – |  |  |  |  |  |  |  |

**table S4** Environmental niche modeling (ENM) for the genus *Oropodisma* and the species *O. macedonica*, *O. willemsei*, and *O. chelmosi*. Table shows the parameters of the best models and the variables retained sorted from higher to lower values of permutation importance. Variables in bold are those that cumulatively contributed > 50% to the model based on the permutation importance statistic.

| Taxon | *n* | *FC* | *RM* | AUC_TEST_ | OR_MTP_ | MTSS | Environmental variables |
| --- | --- | --- | --- | --- | --- | --- | --- |
| Genus: *Oropodisma* | 84 | LQP | 1 | 0.981 | 0.107 | 0.118 | **BIO10**, BIO18, BIO15, BIO13, BIO3, BIO6, BIO8, BIO9, BIO7 |
| Species: *O. macedonica* | 24 | LQHPT | 2 | 0.985 | 0.332 | 0.273 | **BIO8**, BIO18, BIO7, BIO6, BIO13, BIO10, BIO3, BIO9, BIO15 |
| Species: *O. willemsei* | 10 | LQP | 1.5 | 0.974 | 0.208 | 0.524 | **BIO6**, BIO18, BIO9, BIO15, BIO3, BIO10, BIO6 |
| Species: *O. chelmosi* | 11 | LQH | 1.5 | 0.999 | 0.000 | 0.000 | **BIO3**, **BIO8,** BIO15, BIO18, BIO10, BIO7 |

*n*, number of occurrence records used for ENM; *FC*, feature class for variable transformation; *RM*, regularization multiplier; AUC_TEST_, area under the curve of the receiver-operating characteristic plot on the testing data; OR_MTP_, omission rate based on the minimum training presence threshold; MTSS, maximum training sensitivity plus specificity logistic threshold for presence/absence

**table S5** Univariate matrix regressions with randomization for genetic differentiation (*F*_ST_) between populations of *Oropodisma macedonica* in relation to (i) geographical distance, (ii) weighted topographical distance, and resistance distances defined by (iii) a flat landscape (IBR_FLAT_; i.e., all cells have equal resistance value = 1), (iv) contemporary habitat suitability (IBR_CURRENT_) and (v) habitat suitability stability from the LGM to present (IBR_STABILITY_). *R*^2^, coefficient of determination; β, regression coefficient; *t*, *t*-statistic; *p*, significance level

| Variable | *R*^2^ | β | *t* | *p* |
| --- | --- | --- | --- | --- |
| Geographical distance | 0.313 | 0.511 | 4.43 | 0.001 |
| Weighted topographic distance | 0.218 | 0.440 | 3.47 | 0.005 |
| IBR_FLAT_ | 0.300 | 0.870 | 4.29 | 0.001 |
| IBR_CURRENT_ | 0.017 | 0.135 | 0.87 | 0.192 |
| IBR_STABILITY_ | 0.353 | 0.545 | 4.84 | <0.001 |

**TABLE S6** Pairwise Euclidean distances between least-squares means for shape variation of the phallus apex of males (below the diagonal) and their corresponding *q*-values (above the diagonal) for the analyzed populations of *Oropodisma* (*n* ≥ 2). Statistically significant values after false discovery rate adjustment (FDR) to control for multiple tests (FDR of 5%, *q* < 0.05) are indicated in bold. Population codes as described in Table 1.

| Code | LJUB | POPO | MAVR | GALI | VALA | GRAM | SMOL | VASI | MAVV | TYMF | KAKA | TZOU | AVGO | KARA | KAZA | GAVR | AGRA | TRIA | TYMP | KALI | OXIA | VARD | OITI | PIRG | PARS | ERYM | KYLL | PANA | CHEL | MAEN | TAYG |
| --- | --- | --- | --- | --- | --- | --- | --- | --- | --- | --- | --- | --- | --- | --- | --- | --- | --- | --- | --- | --- | --- | --- | --- | --- | --- | --- | --- | --- | --- | --- | --- |
| LJUB | – | 0.966 | 0.997 | 0.996 | 0.966 | 0.997 | 0.967 | 0.691 | 0.758 | 0.967 | 0.982 | 0.844 | 0.705 | 0.615 | 0.638 | 0.738 | 0.009 | 0.717 | 0.207 | 0.434 | 0.123 | 0.166 | 0.536 | 0.273 | 0.094 | 0.016 | 0.009 | 0.016 | 0.009 | 0.068 | 0.434 |
| POPO | 0.097 | – | 0.928 | 0.890 | 0.745 | 0.928 | 0.948 | 0.648 | 0.804 | 0.833 | 0.833 | 0.833 | 0.520 | 0.481 | 0.576 | 0.618 | 0.009 | 0.612 | 0.173 | 0.319 | 0.061 | 0.096 | 0.332 | 0.170 | 0.080 | 0.021 | 0.009 | 0.009 | 0.009 | 0.043 | 0.397 |
| MAVR | 0.060 | 0.108 | – | 0.997 | 0.997 | 0.997 | 0.972 | 0.662 | 0.789 | 0.983 | 0.973 | 0.778 | 0.730 | 0.554 | 0.639 | 0.755 | 0.009 | 0.763 | 0.269 | 0.484 | 0.125 | 0.180 | 0.489 | 0.252 | 0.102 | 0.040 | 0.021 | 0.009 | 0.021 | 0.073 | 0.319 |
| GALI | 0.076 | 0.115 | 0.054 | – | 0.966 | 0.988 | 0.967 | 0.644 | 0.702 | 0.992 | 0.973 | 0.757 | 0.699 | 0.558 | 0.648 | 0.771 | 0.009 | 0.705 | 0.247 | 0.484 | 0.136 | 0.190 | 0.517 | 0.279 | 0.080 | 0.032 | 0.009 | 0.009 | 0.021 | 0.056 | 0.331 |
| VALA | 0.092 | 0.145 | 0.073 | 0.094 | – | 0.997 | 0.777 | 0.778 | 0.847 | 0.928 | 0.966 | 0.738 | 0.909 | 0.684 | 0.736 | 0.943 | 0.009 | 0.928 | 0.423 | 0.648 | 0.170 | 0.283 | 0.684 | 0.434 | 0.113 | 0.056 | 0.021 | 0.025 | 0.021 | 0.079 | 0.331 |
| GRAM | 0.062 | 0.108 | 0.056 | 0.081 | 0.066 | – | 0.928 | 0.755 | 0.914 | 0.967 | 0.957 | 0.778 | 0.755 | 0.607 | 0.671 | 0.810 | 0.009 | 0.810 | 0.306 | 0.533 | 0.123 | 0.196 | 0.571 | 0.306 | 0.096 | 0.029 | 0.009 | 0.016 | 0.009 | 0.061 | 0.353 |
| SMOL | 0.092 | 0.103 | 0.092 | 0.092 | 0.136 | 0.110 | – | 0.614 | 0.611 | 0.997 | 0.893 | 0.699 | 0.539 | 0.408 | 0.510 | 0.638 | 0.009 | 0.639 | 0.143 | 0.314 | 0.073 | 0.099 | 0.379 | 0.150 | 0.040 | 0.009 | 0.009 | 0.009 | 0.009 | 0.050 | 0.397 |
| VASI | 0.161 | 0.177 | 0.167 | 0.175 | 0.141 | 0.146 | 0.188 | – | 0.684 | 0.624 | 0.684 | 0.662 | 0.771 | 0.667 | 0.778 | 0.770 | 0.009 | 0.667 | 0.306 | 0.619 | 0.191 | 0.283 | 0.670 | 0.367 | 0.197 | 0.056 | 0.025 | 0.025 | 0.009 | 0.096 | 0.513 |
| MAVV | 0.143 | 0.134 | 0.135 | 0.155 | 0.125 | 0.111 | 0.188 | 0.164 | – | 0.623 | 0.702 | 0.711 | 0.691 | 0.648 | 0.715 | 0.662 | 0.009 | 0.747 | 0.397 | 0.520 | 0.169 | 0.206 | 0.486 | 0.322 | 0.202 | 0.071 | 0.034 | 0.016 | 0.029 | 0.096 | 0.347 |
| TYMF | 0.093 | 0.125 | 0.082 | 0.075 | 0.110 | 0.094 | 0.065 | 0.176 | 0.186 | – | 0.943 | 0.648 | 0.639 | 0.477 | 0.580 | 0.745 | 0.009 | 0.684 | 0.188 | 0.380 | 0.101 | 0.165 | 0.429 | 0.217 | 0.068 | 0.029 | 0.016 | 0.009 | 0.009 | 0.050 | 0.342 |
| KAKA | 0.087 | 0.128 | 0.092 | 0.086 | 0.097 | 0.100 | 0.116 | 0.162 | 0.155 | 0.108 | – | 0.967 | 0.847 | 0.729 | 0.755 | 0.943 | 0.009 | 0.779 | 0.342 | 0.639 | 0.194 | 0.279 | 0.705 | 0.380 | 0.110 | 0.034 | 0.009 | 0.009 | 0.016 | 0.068 | 0.461 |
| TZOU | 0.124 | 0.127 | 0.137 | 0.143 | 0.144 | 0.138 | 0.153 | 0.168 | 0.152 | 0.167 | 0.091 | – | 0.705 | 0.684 | 0.684 | 0.711 | 0.009 | 0.648 | 0.246 | 0.517 | 0.150 | 0.211 | 0.634 | 0.266 | 0.101 | 0.043 | 0.009 | 0.016 | 0.009 | 0.059 | 0.533 |
| AVGO | 0.160 | 0.200 | 0.154 | 0.159 | 0.112 | 0.145 | 0.198 | 0.143 | 0.165 | 0.176 | 0.122 | 0.155 | – | 0.966 | 0.960 | 0.997 | 0.009 | 0.967 | 0.687 | 0.997 | 0.520 | 0.702 | 0.997 | 0.810 | 0.228 | 0.094 | 0.034 | 0.032 | 0.032 | 0.102 | 0.474 |
| KARA | 0.189 | 0.217 | 0.192 | 0.192 | 0.162 | 0.180 | 0.227 | 0.167 | 0.170 | 0.215 | 0.148 | 0.163 | 0.095 | – | 0.997 | 0.918 | 0.009 | 0.833 | 0.635 | 0.928 | 0.493 | 0.653 | 0.928 | 0.687 | 0.229 | 0.136 | 0.040 | 0.056 | 0.043 | 0.154 | 0.648 |
| KAZA | 0.184 | 0.204 | 0.185 | 0.185 | 0.157 | 0.174 | 0.215 | 0.145 | 0.166 | 0.201 | 0.154 | 0.175 | 0.101 | 0.065 | – | 0.928 | 0.009 | 0.833 | 0.638 | 0.914 | 0.513 | 0.648 | 0.901 | 0.669 | 0.301 | 0.152 | 0.043 | 0.043 | 0.040 | 0.156 | 0.648 |
| GAVR | 0.144 | 0.185 | 0.139 | 0.137 | 0.104 | 0.130 | 0.174 | 0.143 | 0.167 | 0.147 | 0.104 | 0.153 | 0.055 | 0.112 | 0.112 | – | 0.009 | 0.957 | 0.612 | 0.948 | 0.367 | 0.580 | 0.978 | 0.705 | 0.169 | 0.080 | 0.032 | 0.032 | 0.034 | 0.094 | 0.472 |
| AGRA | **1.112** | **1.124** | **1.114** | **1.118** | **1.075** | **1.095** | **1.168** | **1.021** | **1.036** | **1.152** | **1.088** | **1.070** | **1.015** | **0.985** | **0.988** | **1.046** | – | 0.009 | 0.009 | 0.009 | 0.009 | 0.009 | 0.009 | 0.009 | 0.009 | 0.009 | 0.009 | 0.009 | 0.009 | 0.009 | 0.009 |
| TRIA | 0.152 | 0.186 | 0.138 | 0.151 | 0.105 | 0.128 | 0.178 | 0.166 | 0.148 | 0.159 | 0.138 | 0.174 | 0.094 | 0.128 | 0.132 | 0.100 | **1.068** | – | 0.662 | 0.810 | 0.302 | 0.481 | 0.729 | 0.648 | 0.113 | 0.073 | 0.025 | 0.021 | 0.021 | 0.082 | 0.397 |
| TYMP | 0.281 | 0.310 | 0.264 | 0.266 | 0.218 | 0.252 | 0.320 | 0.252 | 0.224 | 0.292 | 0.247 | 0.269 | 0.164 | 0.176 | 0.188 | 0.184 | **0.973** | 0.166 | – | 0.770 | 0.667 | 0.691 | 0.574 | 0.891 | 0.176 | 0.173 | 0.034 | 0.025 | 0.034 | 0.141 | 0.194 |
| KALI | 0.219 | 0.254 | 0.214 | 0.213 | 0.173 | 0.202 | 0.257 | 0.184 | 0.207 | 0.232 | 0.180 | 0.205 | 0.070 | 0.107 | 0.116 | 0.103 | **0.976** | 0.134 | 0.138 | – | 0.745 | 0.962 | 0.983 | 0.986 | 0.266 | 0.145 | 0.040 | 0.029 | 0.034 | 0.116 | 0.397 |
| OXIA | 0.350 | 0.387 | 0.344 | 0.340 | 0.298 | 0.332 | 0.395 | 0.296 | 0.318 | 0.367 | 0.306 | 0.324 | 0.206 | 0.205 | 0.221 | 0.236 | **0.872** | 0.264 | 0.170 | 0.148 | – | 0.967 | 0.580 | 0.829 | 0.309 | 0.235 | 0.059 | 0.038 | 0.038 | 0.169 | 0.207 |
| VARD | 0.311 | 0.347 | 0.305 | 0.301 | 0.261 | 0.293 | 0.343 | 0.259 | 0.294 | 0.315 | 0.264 | 0.290 | 0.160 | 0.174 | 0.184 | 0.184 | **0.937** | 0.216 | 0.158 | 0.102 | 0.093 | – | 0.758 | 0.967 | 0.198 | 0.163 | 0.032 | 0.032 | 0.029 | 0.129 | 0.272 |
| OITI | 0.198 | 0.238 | 0.202 | 0.202 | 0.164 | 0.191 | 0.235 | 0.164 | 0.210 | 0.215 | 0.154 | 0.178 | 0.071 | 0.108 | 0.121 | 0.086 | **0.989** | 0.152 | 0.193 | 0.078 | 0.188 | 0.140 | – | 0.778 | 0.225 | 0.082 | 0.029 | 0.032 | 0.016 | 0.096 | 0.517 |
| PIRG | 0.274 | 0.312 | 0.264 | 0.264 | 0.217 | 0.253 | 0.312 | 0.236 | 0.253 | 0.282 | 0.235 | 0.264 | 0.128 | 0.169 | 0.176 | 0.151 | **0.968** | 0.175 | 0.114 | 0.084 | 0.128 | 0.088 | 0.135 | – | 0.190 | 0.105 | 0.038 | 0.034 | 0.021 | 0.112 | 0.240 |
| PARS | 0.367 | 0.380 | 0.366 | 0.374 | 0.329 | 0.350 | **0.423** | 0.289 | 0.301 | 0.405 | 0.352 | 0.345 | 0.284 | 0.276 | 0.268 | 0.312 | **0.780** | 0.339 | 0.300 | 0.270 | 0.254 | 0.291 | 0.279 | 0.285 | – | 0.127 | 0.096 | 0.059 | 0.054 | 0.207 | 0.189 |
| ERYM | **0.457** | **0.490** | **0.451** | **0.454** | 0.410 | **0.446** | **0.499** | 0.409 | 0.409 | **0.481** | **0.431** | **0.439** | 0.358 | 0.321 | 0.328 | 0.384 | **0.846** | 0.374 | 0.300 | 0.334 | 0.279 | 0.311 | 0.359 | 0.329 | 0.335 | – | 0.298 | 0.184 | 0.174 | 0.313 | 0.150 |
| KYLL | **0.565** | **0.584** | **0.558** | **0.563** | **0.527** | **0.560** | **0.614** | **0.524** | **0.506** | **0.601** | **0.547** | **0.535** | **0.491** | **0.458** | **0.461** | **0.521** | **0.785** | **0.516** | **0.446** | **0.472** | 0.416 | **0.469** | **0.495** | **0.477** | 0.363 | 0.256 | – | 0.380 | 0.255 | 0.576 | 0.038 |
| PANA | **0.556** | **0.566** | **0.547** | **0.544** | **0.523** | **0.550** | **0.587** | **0.505** | **0.505** | **0.574** | **0.540** | **0.540** | **0.488** | 0.451 | **0.439** | **0.503** | **0.874** | **0.507** | **0.462** | **0.473** | **0.443** | **0.473** | **0.492** | **0.480** | 0.380 | 0.306 | 0.241 | – | 0.948 | 0.997 | 0.076 |
| CHEL | **0.543** | **0.554** | **0.537** | **0.533** | **0.515** | **0.543** | **0.570** | **0.504** | **0.505** | **0.560** | **0.526** | **0.528** | **0.479** | **0.439** | **0.430** | **0.495** | **0.922** | **0.495** | **0.466** | **0.468** | **0.448** | **0.472** | **0.484** | **0.480** | 0.405 | 0.307 | 0.261 | 0.100 | – | 0.966 | 0.082 |
| MAEN | 0.570 | **0.582** | 0.562 | 0.558 | 0.536 | 0.565 | 0.603 | 0.521 | 0.522 | 0.588 | 0.556 | 0.560 | 0.503 | 0.468 | 0.453 | 0.520 | **0.870** | 0.522 | 0.472 | 0.487 | 0.452 | 0.487 | 0.510 | 0.492 | 0.391 | 0.311 | 0.238 | 0.091 | 0.117 | – | 0.113 |
| TAYG | 0.277 | 0.284 | 0.306 | 0.307 | 0.302 | 0.293 | 0.284 | 0.256 | 0.303 | 0.301 | 0.263 | 0.252 | 0.263 | 0.219 | 0.227 | 0.260 | **1.053** | 0.278 | 0.373 | 0.282 | 0.372 | 0.326 | 0.246 | 0.343 | 0.395 | 0.444 | **0.577** | 0.544 | 0.517 | 0.567 | – |

**TABLE S7** Pairwise Euclidean distances between least-squares means for shape variation of the furculae of males (below the diagonal) and their corresponding *q*-values (above the diagonal) for the analyzed populations of *Oropodisma* (*n* ≥ 2). Statistically significant values after false discovery rate adjustment (FDR) to control for multiple tests (FDR of 5%, *q* < 0.05) are indicated in bold. Population codes as described in Table 1.

| Code | LJUB | POPO | MAVR | GALI | VALA | GRAM | SMOL | VASI | MAVV | TYMF | KAKA | TZOU | AVGO | KARA | KAZA | GAVR | AGRA | TRIA | TYMP | KALI | OXIA | VARD | OITI | PIRG | PARS | ERYM | KYLL | PANA | CHEL | MAEN | TAYG |
| --- | --- | --- | --- | --- | --- | --- | --- | --- | --- | --- | --- | --- | --- | --- | --- | --- | --- | --- | --- | --- | --- | --- | --- | --- | --- | --- | --- | --- | --- | --- | --- |
| LJUB | – | 0.678 | 0.439 | 0.142 | 0.156 | 0.626 | 0.397 | 0.087 | 0.376 | 0.627 | 0.030 | 0.082 | 0.087 | 0.036 | 0.246 | 0.027 | 0.006 | 0.016 | 0.144 | 0.164 | 0.552 | 0.142 | 0.191 | 0.019 | 0.053 | 0.211 | 0.080 | 0.006 | 0.141 | 0.247 | 0.167 |
| POPO | 0.067 | – | 0.711 | 0.462 | 0.622 | 0.850 | 0.786 | 0.191 | 0.482 | 0.707 | 0.142 | 0.186 | 0.430 | 0.156 | 0.648 | 0.142 | 0.006 | 0.122 | 0.380 | 0.212 | 0.976 | 0.382 | 0.625 | 0.103 | 0.163 | 0.036 | 0.013 | 0.006 | 0.042 | 0.147 | 0.181 |
| MAVR | 0.084 | 0.065 | – | 0.688 | 0.552 | 0.823 | 0.823 | 0.492 | 0.691 | 0.583 | 0.107 | 0.095 | 0.611 | 0.091 | 0.639 | 0.091 | 0.006 | 0.024 | 0.446 | 0.162 | 0.543 | 0.526 | 0.468 | 0.040 | 0.374 | 0.062 | 0.034 | 0.006 | 0.032 | 0.212 | 0.201 |
| GALI | 0.116 | 0.081 | 0.065 | – | 0.865 | 0.745 | 0.914 | 0.443 | 0.546 | 0.353 | 0.349 | 0.232 | 0.977 | 0.392 | 0.952 | 0.430 | 0.006 | 0.113 | 0.632 | 0.103 | 0.552 | 0.663 | 0.819 | 0.059 | 0.492 | 0.034 | 0.016 | 0.006 | 0.021 | 0.071 | 0.237 |
| VALA | 0.111 | 0.070 | 0.077 | 0.053 | – | 0.867 | 0.967 | 0.233 | 0.393 | 0.430 | 0.430 | 0.398 | 0.691 | 0.552 | 0.879 | 0.636 | 0.006 | 0.223 | 0.575 | 0.086 | 0.741 | 0.510 | 0.942 | 0.089 | 0.351 | 0.016 | 0.006 | 0.006 | 0.013 | 0.038 | 0.191 |
| GRAM | 0.071 | 0.053 | 0.055 | 0.062 | 0.054 | – | 0.984 | 0.411 | 0.710 | 0.833 | 0.203 | 0.286 | 0.632 | 0.230 | 0.865 | 0.212 | 0.006 | 0.102 | 0.632 | 0.233 | 0.843 | 0.624 | 0.710 | 0.084 | 0.351 | 0.089 | 0.032 | 0.006 | 0.036 | 0.129 | 0.236 |
| SMOL | 0.091 | 0.058 | 0.057 | 0.048 | 0.041 | 0.034 | – | 0.546 | 0.775 | 0.742 | 0.200 | 0.237 | 0.757 | 0.276 | 0.875 | 0.271 | 0.006 | 0.098 | 0.777 | 0.243 | 0.875 | 0.807 | 0.810 | 0.161 | 0.583 | 0.044 | 0.019 | 0.006 | 0.024 | 0.070 | 0.156 |
| VASI | 0.127 | 0.111 | 0.081 | 0.082 | 0.103 | 0.087 | 0.077 | – | 0.896 | 0.514 | 0.016 | 0.019 | 0.380 | 0.021 | 0.275 | 0.027 | 0.006 | 0.006 | 0.632 | 0.377 | 0.209 | 0.901 | 0.175 | 0.126 | 0.839 | 0.019 | 0.009 | 0.006 | 0.009 | 0.047 | 0.046 |
| MAVV | 0.092 | 0.083 | 0.065 | 0.077 | 0.090 | 0.064 | 0.061 | 0.050 | – | 0.873 | 0.038 | 0.040 | 0.378 | 0.042 | 0.435 | 0.049 | 0.006 | 0.013 | 0.742 | 0.605 | 0.552 | 0.907 | 0.311 | 0.195 | 0.624 | 0.049 | 0.016 | 0.006 | 0.021 | 0.089 | 0.068 |
| TYMF | 0.072 | 0.064 | 0.074 | 0.092 | 0.087 | 0.056 | 0.063 | 0.080 | 0.052 | – | 0.034 | 0.047 | 0.265 | 0.057 | 0.413 | 0.036 | 0.006 | 0.013 | 0.749 | 0.742 | 0.714 | 0.710 | 0.386 | 0.328 | 0.436 | 0.032 | 0.019 | 0.006 | 0.019 | 0.088 | 0.053 |
| KAKA | **0.147** | 0.116 | 0.121 | 0.091 | 0.084 | 0.106 | 0.106 | **0.163** | **0.148** | **0.149** | – | 0.916 | 0.325 | 0.916 | 0.622 | 0.877 | 0.006 | 0.691 | 0.068 | 0.009 | 0.174 | 0.047 | 0.458 | 0.009 | 0.021 | 0.009 | 0.009 | 0.006 | 0.013 | 0.046 | 0.615 |
| TZOU | 0.130 | 0.110 | 0.124 | 0.106 | 0.086 | 0.101 | 0.106 | **0.169** | **0.149** | **0.142** | 0.048 | – | 0.195 | 0.658 | 0.417 | 0.590 | 0.006 | 0.521 | 0.046 | 0.006 | 0.203 | 0.044 | 0.381 | 0.006 | 0.009 | 0.016 | 0.006 | 0.009 | 0.009 | 0.034 | 0.636 |
| AVGO | 0.124 | 0.084 | 0.071 | 0.040 | 0.067 | 0.071 | 0.061 | 0.091 | 0.090 | 0.101 | 0.094 | 0.110 | – | 0.368 | 0.877 | 0.380 | 0.006 | 0.113 | 0.473 | 0.071 | 0.514 | 0.583 | 0.617 | 0.044 | 0.420 | 0.021 | 0.009 | 0.006 | 0.009 | 0.088 | 0.180 |
| KARA | **0.152** | 0.112 | 0.126 | 0.086 | 0.076 | 0.107 | 0.101 | **0.160** | **0.145** | 0.142 | 0.045 | 0.069 | 0.094 | – | 0.688 | 0.984 | 0.006 | 0.678 | 0.116 | 0.009 | 0.217 | 0.064 | 0.710 | 0.009 | 0.036 | 0.006 | 0.006 | 0.006 | 0.006 | 0.032 | 0.329 |
| KAZA | 0.108 | 0.073 | 0.073 | 0.044 | 0.053 | 0.057 | 0.055 | 0.106 | 0.091 | 0.093 | 0.076 | 0.093 | 0.054 | 0.069 | – | 0.627 | 0.006 | 0.329 | 0.546 | 0.128 | 0.691 | 0.502 | 0.938 | 0.047 | 0.273 | 0.044 | 0.034 | 0.006 | 0.036 | 0.118 | 0.343 |
| GAVR | **0.160** | 0.117 | 0.127 | 0.085 | 0.072 | 0.108 | 0.098 | **0.152** | **0.142** | **0.143** | 0.050 | 0.074 | 0.090 | 0.036 | 0.075 | – | 0.006 | 0.643 | 0.121 | 0.006 | 0.210 | 0.098 | 0.632 | 0.019 | 0.053 | 0.009 | 0.006 | 0.006 | 0.006 | 0.027 | 0.255 |
| AGRA | **0.199** | **0.205** | **0.244** | **0.253** | **0.243** | **0.226** | **0.243** | **0.298** | **0.262** | **0.241** | **0.221** | **0.211** | **0.251** | **0.222** | **0.221** | **0.242** | – | 0.006 | 0.006 | 0.006 | 0.006 | 0.006 | 0.006 | 0.006 | 0.006 | 0.006 | 0.006 | 0.006 | 0.006 | 0.019 | 0.030 |
| TRIA | **0.165** | 0.126 | **0.151** | 0.125 | 0.105 | 0.128 | 0.127 | **0.191** | **0.174** | **0.167** | 0.065 | 0.079 | 0.122 | 0.068 | 0.101 | 0.069 | **0.199** | – | 0.016 | 0.009 | 0.162 | 0.016 | 0.242 | 0.006 | 0.009 | 0.006 | 0.006 | 0.006 | 0.009 | 0.016 | 0.237 |
| TYMP | 0.114 | 0.092 | 0.084 | 0.070 | 0.076 | 0.069 | 0.061 | 0.070 | 0.062 | 0.061 | 0.137 | **0.139** | 0.083 | 0.124 | 0.084 | 0.121 | **0.270** | **0.162** | – | 0.552 | 0.484 | 0.938 | 0.573 | 0.378 | 0.873 | 0.013 | 0.009 | 0.006 | 0.013 | 0.042 | 0.051 |
| KALI | 0.113 | 0.107 | 0.111 | 0.124 | 0.130 | 0.105 | 0.104 | 0.091 | 0.072 | 0.062 | **0.194** | **0.190** | 0.131 | **0.182** | 0.131 | **0.181** | **0.270** | **0.207** | 0.076 | – | 0.248 | 0.547 | 0.070 | 0.572 | 0.383 | 0.013 | 0.006 | 0.006 | 0.006 | 0.040 | 0.021 |
| OXIA | 0.078 | 0.038 | 0.078 | 0.078 | 0.064 | 0.054 | 0.051 | 0.107 | 0.077 | 0.064 | 0.114 | 0.108 | 0.082 | 0.106 | 0.070 | 0.107 | **0.211** | 0.118 | 0.083 | 0.102 | – | 0.546 | 0.694 | 0.212 | 0.231 | 0.027 | 0.006 | 0.006 | 0.024 | 0.057 | 0.119 |
| VARD | 0.118 | 0.087 | 0.077 | 0.067 | 0.080 | 0.071 | 0.058 | 0.048 | 0.047 | 0.064 | **0.141** | **0.146** | 0.075 | 0.133 | 0.084 | 0.124 | **0.273** | **0.162** | 0.047 | 0.078 | 0.078 | – | 0.429 | 0.355 | 0.922 | 0.006 | 0.006 | 0.006 | 0.006 | 0.049 | 0.053 |
| OITI | 0.111 | 0.070 | 0.082 | 0.056 | 0.042 | 0.063 | 0.057 | 0.114 | 0.096 | 0.089 | 0.081 | 0.089 | 0.071 | 0.065 | 0.047 | 0.069 | **0.229** | 0.103 | 0.076 | 0.129 | 0.065 | 0.085 | – | 0.087 | 0.227 | 0.024 | 0.013 | 0.006 | 0.009 | 0.044 | 0.217 |
| PIRG | **0.156** | 0.125 | **0.142** | 0.140 | 0.130 | 0.131 | 0.119 | 0.120 | 0.110 | 0.097 | **0.198** | **0.197** | **0.144** | **0.178** | **0.145** | **0.171** | **0.290** | **0.198** | 0.093 | 0.075 | 0.110 | 0.091 | 0.130 | – | 0.336 | 0.006 | 0.006 | 0.006 | 0.006 | 0.006 | 0.009 |
| PARS | 0.141 | 0.114 | 0.091 | 0.081 | 0.094 | 0.094 | 0.076 | 0.054 | 0.072 | 0.086 | **0.158** | **0.165** | 0.087 | **0.150** | 0.106 | 0.139 | **0.304** | **0.182** | 0.053 | 0.089 | 0.105 | 0.047 | 0.104 | 0.097 | – | 0.009 | 0.006 | 0.006 | 0.006 | 0.021 | 0.034 |
| ERYM | 0.107 | **0.146** | 0.133 | **0.151** | **0.168** | 0.127 | **0.149** | **0.163** | **0.142** | **0.147** | **0.172** | **0.164** | **0.160** | **0.187** | **0.148** | **0.198** | **0.226** | **0.209** | **0.168** | **0.181** | **0.158** | **0.170** | **0.166** | **0.237** | **0.193** | – | 0.806 | 0.156 | 0.681 | 0.457 | 0.319 |
| KYLL | 0.131 | **0.162** | **0.147** | **0.167** | **0.185** | **0.149** | **0.170** | **0.186** | **0.167** | **0.169** | **0.181** | **0.177** | **0.170** | **0.196** | **0.160** | **0.212** | **0.223** | **0.219** | **0.188** | **0.203** | **0.179** | **0.194** | **0.182** | **0.259** | **0.212** | 0.057 | – | 0.309 | 0.865 | 0.694 | 0.247 |
| PANA | **0.183** | **0.214** | **0.213** | **0.226** | **0.233** | **0.203** | **0.227** | **0.259** | **0.236** | **0.230** | **0.203** | **0.191** | **0.229** | **0.223** | **0.210** | **0.241** | **0.203** | **0.234** | **0.249** | **0.271** | **0.229** | **0.258** | **0.228** | **0.318** | **0.282** | 0.116 | 0.097 | – | 0.380 | 0.164 | 0.247 |
| CHEL | 0.118 | **0.146** | **0.144** | **0.167** | **0.178** | **0.143** | **0.165** | **0.191** | **0.168** | **0.166** | **0.171** | **0.163** | **0.168** | **0.187** | **0.154** | **0.202** | **0.187** | **0.199** | **0.193** | **0.204** | **0.165** | **0.193** | **0.176** | **0.254** | **0.218** | 0.067 | 0.052 | 0.092 | – | 0.800 | 0.338 |
| MAEN | 0.136 | 0.154 | 0.141 | 0.175 | **0.193** | 0.160 | 0.177 | **0.189** | 0.169 | 0.172 | **0.198** | **0.199** | 0.171 | **0.210** | 0.166 | **0.224** | **0.213** | **0.224** | **0.198** | **0.196** | 0.176 | **0.195** | **0.191** | **0.249** | **0.214** | 0.113 | 0.085 | 0.152 | 0.078 | – | 0.247 |
| TAYG | 0.150 | 0.149 | 0.142 | 0.139 | 0.144 | 0.137 | 0.151 | **0.194** | 0.177 | 0.180 | 0.093 | 0.092 | 0.144 | 0.126 | 0.129 | 0.136 | **0.215** | 0.136 | 0.184 | **0.229** | 0.160 | 0.186 | 0.141 | **0.255** | **0.207** | 0.128 | 0.133 | 0.135 | 0.121 | 0.165 | – |

**TABLE S8** Pairwise Euclidean distances between least-squares means for shape variation of the phallus apex of males (below the diagonal) and their corresponding *q*-values (above the diagonal) for the analyzed species of *Oropodisma*. Statistically significant values after false discovery rate adjustment (FDR) to control for multiple tests (FDR of 5%, *q* < 0.05) are indicated in bold.

|  |  | MAC | TZO | KAR | AGR | LAG | TYM | WIL | PAR | ERY | KYL | CHE | TAY |
| --- | --- | --- | --- | --- | --- | --- | --- | --- | --- | --- | --- | --- | --- |
| *O. macedonica* | MAC | – | 0.468 | 0.063 | 0.004 | 0.468 | 0.047 | 0.004 | 0.022 | 0.007 | 0.004 | 0.004 | 0.121 |
| *O. tzoumerkae* | TZO | 0.093 | – | 0.287 | 0.004 | 0.468 | 0.098 | 0.017 | 0.026 | 0.009 | 0.007 | 0.004 | 0.233 |
| *O. karavica* | KAR | 0.140 | 0.128 | – | 0.004 | 0.779 | 0.264 | 0.176 | 0.043 | 0.018 | 0.011 | 0.004 | 0.233 |
| *O. agrafae* | AGR | **1.099** | **1.078** | **1.008** | – | 0.004 | 0.004 | 0.004 | 0.004 | 0.004 | 0.004 | 0.004 | 0.004 |
| *O. lagrecai* | LAG | 0.129 | 0.150 | 0.099 | **1.068** | – | 0.472 | 0.233 | 0.048 | 0.040 | 0.011 | 0.004 | 0.250 |
| *O. tymphrestosi* | TYM | **0.257** | 0.254 | 0.169 | **0.973** | 0.166 | – | 0.423 | 0.103 | 0.094 | 0.017 | 0.009 | 0.106 |
| *O. willemsei* | WIL | **0.248** | **0.229** | 0.123 | **0.946** | 0.178 | 0.136 | – | 0.048 | 0.025 | 0.011 | 0.004 | 0.106 |
| *O. parnassica* | PAR | **0.351** | **0.345** | **0.281** | **0.780** | **0.339** | 0.300 | **0.265** | – | 0.069 | 0.043 | 0.017 | 0.106 |
| *O. erymanthosi* | ERY | **0.444** | **0.432** | **0.345** | **0.846** | **0.374** | 0.300 | **0.314** | 0.335 | – | 0.193 | 0.045 | 0.081 |
| *O. kyllinii* | KYL | **0.555** | **0.539** | **0.481** | **0.785** | **0.516** | **0.446** | **0.460** | **0.363** | 0.256 | – | 0.114 | 0.023 |
| *O. chelmosi* | CHE | **0.536** | **0.533** | **0.465** | **0.892** | **0.501** | **0.462** | **0.464** | **0.388** | **0.302** | 0.243 | – | 0.035 |
| *O. taygetosi* | TAY | 0.280 | 0.254 | 0.237 | **1.053** | 0.278 | 0.373 | 0.307 | 0.395 | 0.444 | **0.577** | **0.534** | – |

**TABLE S9** Pairwise Euclidean distances between least-squares means for shape variation of the furculae of males (below the diagonal) and their corresponding *q*-values (above the diagonal) for the analyzed species of *Oropodisma*. Statistically significant values after false discovery rate adjustment (FDR) to control for multiple tests (FDR of 5%, *q* < 0.05) are indicated in bold.

|  |  | MAC | TZO | KAR | AGR | LAG | TYM | WIL | PAR | ERY | KYL | CHE | TAY |
| --- | --- | --- | --- | --- | --- | --- | --- | --- | --- | --- | --- | --- | --- |
| *O. macedonica* | MAC | – | 0.002 | 0.004 | 0.002 | 0.002 | 0.436 | 0.039 | 0.172 | 0.002 | 0.002 | 0.002 | 0.047 |
| *O. tzoumerkae* | TZO | **0.112** | – | 0.218 | 0.002 | 0.711 | 0.008 | 0.002 | 0.002 | 0.002 | 0.004 | 0.002 | 0.535 |
| *O. karavica* | KAR | **0.081** | 0.060 | – | 0.002 | 0.737 | 0.061 | 0.002 | 0.013 | 0.002 | 0.002 | 0.002 | 0.172 |
| *O. agrafae* | AGR | **0.237** | **0.214** | **0.231** | – | 0.002 | 0.002 | 0.002 | 0.002 | 0.002 | 0.002 | 0.002 | 0.017 |
| *O. lagrecai* | LAG | **0.139** | 0.068 | 0.081 | **0.199** | – | 0.005 | 0.002 | 0.004 | 0.002 | 0.002 | 0.002 | 0.194 |
| *O. tymphrestosi* | TYM | 0.059 | **0.136** | 0.096 | **0.270** | **0.162** | – | 0.737 | 0.797 | 0.005 | 0.005 | 0.002 | 0.034 |
| *O. willemsei* | WIL | **0.050** | **0.137** | **0.097** | **0.249** | **0.151** | 0.046 | – | 0.328 | 0.002 | 0.002 | 0.002 | 0.017 |
| *O. parnassica* | PAR | 0.078 | **0.160** | **0.116** | **0.304** | **0.182** | 0.053 | 0.067 | – | 0.002 | 0.002 | 0.002 | 0.018 |
| *O. erymanthosi* | ERY | **0.135** | **0.166** | **0.170** | **0.226** | **0.209** | **0.168** | **0.174** | **0.193** | – | 0.737 | 0.263 | 0.237 |
| *O. kyllinii* | KYL | **0.156** | **0.177** | **0.181** | **0.223** | **0.219** | **0.188** | **0.196** | **0.212** | 0.057 | – | 0.711 | 0.197 |
| *O. chelmosi* | CHE | **0.173** | **0.174** | **0.189** | **0.190** | **0.210** | **0.211** | **0.213** | **0.239** | 0.079 | 0.053 | – | 0.196 |
| *O. taygetosi* | TAY | **0.149** | 0.089 | 0.127 | **0.215** | 0.136 | **0.184** | **0.189** | **0.207** | 0.128 | 0.133 | 0.122 | – |

**Supplementary figures**

**figure S1** Phylogenetic tree inferred with raxml and divergence times estimated using bpp (analysis A00) for the analyzed populations of *Oropodisma*. Bootstrap support values estimated with raxml are indicated on the nodes (* > 95). Node support for the separation of *O. macedonica* populations is generally low and not shown on the tree. Bars on nodes indicate 95% highest posterior densities (HPD) of divergence times estimated considering a genomic mutation rate of 2.8 × 10^−9^ per site per generation and a one-year generation time. Background colors indicate geological divisions of the Quaternary. The taxonomic status of populations before (? = unknown) and after species delimitation analyses in delineate is indicated. Population codes as described in Table 1, with crosses denoting type localities.

**figure S2** Phylogenetic tree inferred with svdquartets and divergence times estimated using bpp (analysis A00) for the analyzed populations of *Oropodisma*. Bootstrap support values estimated with svdquartets are indicated on the nodes (* > 95). Node support for the separation of *O. macedonica* populations is generally low and not shown on the tree. Bars on nodes indicate 95% highest posterior densities (HPD) of divergence times estimated considering a genomic mutation rate of 2.8 × 10^−9^ per site per generation and a one-year generation time. Background colors indicate geological divisions of the Quaternary. The taxonomic status of populations before (? = unknown) and after species delimitation analyses in delineate is indicated. Population codes as described in Table 1, with crosses denoting type localities.

**figure S3** Summary of model fit with phylonetworks. The figure shows the negative log pseudo-likelihood for models with different number of introgression events (*h* from 0 to 5).

**figure S4** Results of structure for (A) *O. macedonica*, (B) *O. tzoumerkae*, (C) *O. karavica*, (D) *O. willemsei*, (E) *O. parnassica*, and (F) *O. chelmosi*. Panels show mean (±SD) log probability of the data (LnPr(X|*K*)) over 10 runs of structure (left axes, black dots and error bars) for each value of *K* and the magnitude of Δ*K* (right axes, open blue triangles).

**FIGURE S5** (A) Principal component analysis (PCA) of genetic variation and (B) results of genetic assignments based on the program structure for *Oropodisma macedonica*. Both analyses are based on a dataset of 10,000 SNPs. In barplots, each individual is represented by a vertical bar partitioned into *K* colored segments showing the individual’s probability of belonging to the cluster with that color; thin vertical black lines separate individuals from different populations. Population codes as described in Table 1.

**FIGURE S6** (A) Principal component analysis (PCA) of genetic variation and (B) results of genetic assignments based on the program structure for *Oropodisma tzoumerkae*. Both analyses are based on a dataset of 6,644 SNPs. In barplots, each individual is represented by a vertical bar partitioned into *K* colored segments showing the individual’s probability of belonging to the cluster with that color; thin vertical black lines separate individuals from different populations. Population codes as described in Table 1.

**FIGURE S7** (A) Principal component analysis (PCA) of genetic variation and (B) results of genetic assignments based on the program structure for *Oropodisma karavica*. Both analyses are based on a dataset of 10,000 SNPs. In barplots, each individual is represented by a vertical bar partitioned into *K* colored segments showing the individual’s probability of belonging to the cluster with that color; thin vertical black lines separate individuals from different populations. Population codes as described in Table 1.

**FIGURE S8** (A) Principal component analysis (PCA) of genetic variation and (B) results of genetic assignments based on the program structure for *Oropodisma willemsei*. Both analyses are based on a dataset of 8,386 SNPs. In barplots, each individual is represented by a vertical bar partitioned into *K* colored segments showing the individual’s probability of belonging to the cluster with that color; thin vertical black lines separate individuals from different populations. Population codes as described in Table 1.

**FIGURE S9** (A) Principal component analysis (PCA) of genetic variation and (B) results of genetic assignments based on the program structure for *Oropodisma parnassica*. Both analyses are based on a dataset of 10,000 SNPs. In barplots, each individual is represented by a vertical bar partitioned into *K* colored segments showing the individual’s probability of belonging to the cluster with that color; thin vertical black lines separate individuals from different populations. Population codes as described in Table 1.

**FIGURE S10** (A) Principal component analysis (PCA) of genetic variation and (B) results of genetic assignments based on the program structure for *Oropodisma chelmosi*. Both analyses are based on a dataset of 10,000 SNPs. In barplots, each individual is represented by a vertical bar partitioned into *K* colored segments showing the individual’s probability of belonging to the cluster with that color; thin vertical black lines separate individuals from different populations. Population codes as described in Table 1.

**FIGURE S11** Extent of climatically suitable habitats for *Oropodisma macedonica* as inferred from projections of the species-specific environmental niche model (ENM) to bioclimatic conditions during the last 22,000 years (i.e., from 1990 CE to the last glacial maximum, LGM) at 100-year time intervals. (A) The availability of suitable habitats at each time interval was calculated as the number of cells where the probability of presence of the species is higher than the maximum training sensitivity plus specificity (MTSS) logistic threshold. (B-F) Maps show the distribution of climatically suitable habitats for *O. macedonica* at five temporal snapshots (red dots in panel A), including (B) the present (0 ka; crosses show occurrence points used for ENM), (D) Holocene Climate Optimum (ca. 10 ka) and (F) LGM (ca. 22 ka).

**FIGURE S12** Extent of climatically suitable habitats for *Oropodisma willemsei* as inferred from projections of the species-specific environmental niche model (ENM) to bioclimatic conditions during the last 22,000 years (i.e., from 1990 CE to the last glacial maximum, LGM) at 100-year time intervals. (A) The availability of suitable habitats at each time interval was calculated as the number of cells where the probability of presence of the species is higher than the maximum training sensitivity plus specificity (MTSS) logistic threshold. (B-F) Maps show the distribution of climatically suitable habitats for *O. willemsei* at five temporal snapshots (red dots in panel A), including (B) the present (0 ka; crosses show occurrence points used for ENM), (D) Holocene Climate Optimum (ca. 10 ka) and (F) LGM (ca. 22 ka).

**FIGURE S13** Extent of climatically suitable habitats for *Oropodisma chelmosi* as inferred from projections of the species-specific environmental niche model (ENM) to bioclimatic conditions during the last 22,000 years (i.e., from 1990 CE to the last glacial maximum, LGM) at 100-year time intervals. (A) The availability of suitable habitats at each time interval was calculated as the number of cells where the probability of presence of the species is higher than the maximum training sensitivity plus specificity (MTSS) logistic threshold. (B-F) Maps show the distribution of climatically suitable habitats for *O. chelmosi* at five temporal snapshots (red dots in panel A), including (B) the present (0 ka; crosses show occurrence points used for ENM), (D) Holocene Climate Optimum (ca. 10 ka) and (F) LGM (ca. 22 ka).

**FIGURE S14** Principal component analyses (PCA) for shape variation of (A) the phallus apex (B) the furculae of males for the analyzed individuals of *Oropodisma*. Population codes as described in Table 1.

**FIGURE S15** Two dimensional phylomorphospace plot for shape variation of (A) the phallus apex (PC1p and PC2p) and (B) the furculae (PC1f and PC2f) of males in *Oropodisma*. Dots show mean values for each species and black lines phylogenetic relationships among them.

**FIGURE S16** Reconstructed evolution of shape variation of (A) the phallus apex (PC1p, PC2p) and (B) the furculae (PC1f and PC2f) of males in *Oropodisma*, estimated using the *contMap* function in the r package ‘phytools’ (Revell, 2012). This function plots reconstructed values for ancestral characters along the edges of the tree.

**Supplementary references**

Adams, D. C., & Otarola‐Castillo, E. (2013). ‘geomorph’: An r package for the collection and analysis of geometric morphometric shape data. *Methods in Ecology and Evolution, 4*(4), 393-399. <https://doi.org/10.1111/2041-210x.12035>

Anderson, M. J. (2001). A new method for non-parametric multivariate analysis of variance. *Austral Ecology 26*(1), 32-46. <https://doi.org/10.1111/j.1442-9993.2001.01070.pp.x>

Barve, N., Barve, V., Jimenez-Valverde, A., Lira-Noriega, A., Maher, S. P., Peterson, A. T., . . . Villalobos, F. (2011). The crucial role of the accessible area in ecological niche modeling and species distribution modeling. *Ecological Modelling, 222*(11), 1810-1819. <https://doi.org/10.1016/j.ecolmodel.2011.02.011>

Bernardes, J. S., Dávila, A. M., Costa, V. S., & Zaverucha, G. (2007). Improving model construction of profile HMMs for remote homology detection through structural alignment. *BMC Bioinformatics, 8*, 435. <https://doi.org/10.1186/1471-2105-8-435>

Bolger, A. M., Lohse, M., & Usadel, B. (2014). trimmomatic: a flexible trimmer for Illumina sequence data. *Bioinformatics, 30*(15), 2114-2120. <https://doi.org/10.1093/bioinformatics/btu170>

Capella-Gutiérrez, S., Silla-Martínez, J. M., & Gabaldón, T. (2009). trimal: a tool for automated alignment trimming in large-scale phylogenetic analyses. *Bioinformatics, 25*(15), 1972-1973. <https://doi.org/10.1093/bioinformatics/btp348>

Chifman, J., & Kubatko, L. (2014). Quartet inference from SNP data under the coalescent model. *Bioinformatics, 30*(23), 3317-3324. <https://doi.org/10.1093/bioinformatics/btu530>

Cigliano, M. M., Braun, H., Eades, D. C., & Otte, D. (2025). Orthoptera Species File (OSF) [retrieved at 15/12/2025]. <http://orthoptera.speciesfile.org>

Collyer, M. L., & Adams, D. C. (2018). ‘RRPP’: An r package for fitting linear models to high-dimensional data using residual randomization. *Methods in Ecology and Evolution, 9*(7), 1772-1779. <https://doi.org/10.1111/2041-210X.13029>

Earl, D. A., & vonHoldt, B. M. (2012). structure harvester: a website and program for visualizing structure output and implementing the Evanno method. *Conservation Genetics Resources, 4*(2), 359-361. <https://doi.org/10.1007/s12686-011-9548-7>

Eaton, D. A. R., & I. Overcast (2020). ipyrad: Interactive assembly and analysis of RADseq datasets. *Bioinformatics 36*(8), 2592-2594. <https://doi.org/10.1093/bioinformatics/btz966>

Eaton, D. A. R., Hipp, A. L., González-Rodríguez, A., & Cavender-Bares, J. (2015). Historical introgression among the American live oaks and the comparative nature of tests for introgression. *Evolution, 69*(10), 2587-2601. <https://doi.org/10.1111/evo.12758>

Evanno, G., Regnaut, S., & Goudet, J. (2005). Detecting the number of clusters of individuals using the software structure: a simulation study. *Molecular Ecology, 14*(8), 2611-2620. <https://doi.org/10.1111/j.1365-294X.2005.02553.x>

Excoffier, L., & Lischer, H. E. L. (2010). arlequin suite ver 3.5: a new series of programs to perform population genetics analyses under Linux and Windows. *Molecular Ecology Resources, 10*(3), 564-567. <https://doi.org/10.1111/j.1755-0998.2010.02847.x>

Flouri, T., Jiao, X. Y., Rannala, B., & Yang, Z. H. (2018). Species tree inference with bpp using genomic sequences and the multispecies coalescent. *Molecular Biology and Evolution, 35*(10), 2585-2593. <https://doi.org/10.1093/molbev/msy147>

Gilbert, K. J., Andrew, R. L., Bock, D. G., Franklin, M. T., Kane, N. C., Moore, J. S., & Vines, T. H. (2012). Recommendations for utilizing and reporting population genetic analyses: The reproducibility of genetic clustering using the program structure. *Molecular Ecology, 21*(20), 4925-4930. <https://doi.org/10.1111/j.1365-294X.2012.05754.x>

González-Serna, M. J., Cordero, P. J., & Ortego, J. (2019). Spatiotemporally explicit demographic modelling supports a joint effect of historical barriers to dispersal and contemporary landscape composition on structuring genomic variation in a red-listed grasshopper. *Molecular Ecology, 28*(9), 2155-2172. <https://doi.org/10.1111/mec.15086>

Huang, J. P., Hill, J. G., Ortego, J., & Knowles, L. L. (2020). Paraphyletic species no more - genomic data resolve a Pleistocene radiation and validate morphological species of the *Melanoplus scudderi* complex (Insecta: Orthoptera). *Systematic Entomology, 45*(3), 594-605. <https://doi.org/10.1111/syen.12415>

Jakobsson, M., & Rosenberg, N. A. (2007). clumpp: a cluster matching and permutation program for dealing with label switching and multimodality in analysis of population structure. *Bioinformatics, 23*(14), 1801-1806. <https://doi.org/10.1093/bioinformatics/btm233>

Janes, J. K., Miller, J. M., Dupuis, J. R., Malenfant, R. M., Gorrell, J. C., Cullingham, C. I., & Andrew, R. L. (2017). The *K*=2 conundrum. *Molecular Ecology, 26*(14), 3594-3602. <https://doi.org/10.1111/mec.14187>

Jombart, T. (2008). *adegenet*: a r package for the multivariate analysis of genetic markers. *Bioinformatics, 24*(11), 1403-1405. <https://doi.org/10.1093/bioinformatics/btn129>

Karger, D. N., Conrad, O., Bohner, J., Kawohl, T., Kreft, H., Soria-Auza, R. W., . . . Kessler, M. (2017). Climatologies at high resolution for the earth's land surface areas. *Scientific Data, 4*, 170122. <https://doi.org/10.1038/sdata.2017.122>

Keightley, P. D., Ness, R. W., Halligan, D. L., & Haddrill, P. R. (2014). Estimation of the spontaneous mutation rate per nucleotide site in a *Drosophila melanogaster* full-sib family. *Genetics, 196*(1), 313-320. <https://doi.org/10.1534/genetics.113.158758>

Keightley, P. D., Pinharanda, A., Ness, R. W., Simpson, F., Dasmahapatra, K. K., Mallet, J., Davey, J. W., & Jiggins, C. D. (2015). Estimation of the spontaneous mutation rate in *Heliconius melpomene*. *Molecular Biology and Evolution, 32*(1), 239-243. <https://doi.org/10.1093/molbev/msu302>

La Greca, M., & Messina, A. (1977). Due nuove specie di *Oropodisma* (Orthoptera: Acridoidea) di Grecia. *Animalia, 3* (1976), 5-16.

Lemonnier-Darcemont, M., Puskás, G., & Darcemont, C. (2015). First overview of the south Albanian Orthoptera fauna. *Articulata, 30*, 63-80.

Lewis, P. O. (2001). A likelihood approach to estimating phylogeny from discrete morphological character data. *Systematic Biology, 50*(6), 913-925. <https://doi.org/10.1080/106351501753462876>

Liu, C. R., Berry, P. M., Dawson, T. P., & Pearson, R. G. (2005). Selecting thresholds of occurrence in the prediction of species distributions. *Ecography, 28*(3), 385-393. <https://doi.org/10.1111/j.0906-7590.2005.03957.x>

Machácková, L., & Fikácek, M. (2014). Catalogue of the type specimens deposited in the Department of Entomology, National Museum, Prague, Czech Republic. *Acta Entomologica Musei Nationalis Pragae, 54*(1), 399-450.

Muscarella, R., Galante, P. J., Soley-Guardia, M., Boria, R. A., Kass, J. M., Uriarte, M., & Anderson, R. P. (2014). ‘ENMeval’: An r package for conducting spatially independent evaluations and estimating optimal model complexity for maxent ecological niche models. *Methods in Ecology and Evolution, 5*(11), 1198-1205. <https://doi.org/10.1111/2041-210x.12261>

McRae, B. H. (2006). Isolation by resistance. *Evolution, 60*(8), 1551-1561. <https://doi.org/10.1111/j.0014-3820.2006.tb00500.x>

McRae, B. H., & Beier, P. (2007). Circuit theory predicts gene flow in plant and animal populations. *Proceedings of the National Academy of Sciences of the United States of America, 104*(50), 19885-19890. <https://doi.org/10.1073/pnas.0706568104>

Nabholz, B. (2024). Incomplete lineage sorting explains the low performance of DNA barcoding in a radiation of four species of Western European grasshoppers (Orthoptera: Acrididae: *Chorthippus*). *Biological Journal of the Linnean Society, 141*(1), 33-50. <https://doi.org/10.1093/biolinnean/blad106>

Noguerales, V., García-Navas, V., Cordero, P. J., & Ortego, J. (2016). The role of environment and core-margin effects on range-wide phenotypic variation in a montane grasshopper. *Journal of Evolutionary Biology, 29*(11), 2129-2142. <https://doi.org/10.1111/jeb.12915>

Ortego, J., Gugger, P. F., & Sork, V. L. (2015). Climatically stable landscapes predict patterns of genetic structure and admixture in the Californian canyon live oak. *Journal of Biogeography, 42*(2), 328-338. <https://doi.org/10.1111/jbi.12419>

Ortego, J., Gugger, P. F., & Sork, V. L. (2018). Genomic data reveal cryptic lineage diversification and introgression in Californian golden cup oaks (section Protobalanus). *New Phytologist, 218*(2), 804-818. <https://doi.org/10.1111/nph.14951>

Padgham, M., & Sumner, M. (2025). *‘*geodist’*: Fast, Dependency-Free Geodesic Distance Calculations*. r package version 0.1.1.001, <https://github.com/hypertidy/geodist>.

Peterson, A. T., Soberón, J., Pearson, R. G., Anderson, R. P., Martínez-Meyer, E., Nakamura, M., & Araújo, M. B. (2011). *Ecological niches and geographic distributions*. Princeton, NJ: Princeton University Press.

Peterson, B. K., Weber, J. N., Kay, E. H., Fisher, H. S., & Hoekstra, H. E. (2012). Double digest RADseq: An inexpensive method for *de novo* SNP discovery and genotyping in model and non-model species. *PLoS One, 7*(5), e37135. <https://doi.org/10.1371/journal.pone.0037135>

Phillips, S. J., Anderson, R. P., & Schapire, R. E. (2006). Maximum entropy modeling of species geographic distributions. *Ecological Modelling, 190*(3-4), 231-259. <https://doi.org/10.1016/j.ecolmodel.2005.03.026>

Phillips, S. J., & Dudik, M. (2008). Modeling of species distributions with maxent: new extensions and a comprehensive evaluation. *Ecography, 31*(2), 161-175. <https://doi.org/10.1111/j.0906-7590.2008.5203.x>

Pritchard, J. K., Stephens, M., & Donnelly, P. (2000). Inference of population structure using multilocus genotype data. *Genetics, 155*(2), 945-959. <https://doi.org/10.1093/genetics/155.2.945>

R Core Team (2025). r*: A language and environment for statistical computing*. Vienna, Austria: R Foundation for Statistical Computing. Available at <https://www.R-project.org/> (accessed September 9, 2025).

Rannala, B., & Yang, Z. H. (2003). Bayes estimation of species divergence times and ancestral population sizes using DNA sequences from multiple loci. *Genetics, 164*(4), 1645-1656. <https://doi.org/10.1093/genetics/164.4.1645>

Rosenberg, N. A. (2004). distruct: a program for the graphical display of population structure. *Molecular Ecology Notes, 4*(1), 137-138. <https://doi.org/10.1046/j.1471-8286.2003.00566.x>

Radosavljevic, A., & Anderson, R. P. (2014). Making better maxent models of species distributions: complexity, overfitting and evaluation. *Journal of Biogeography, 41*(4), 629-643. <https://doi.org/10.1111/jbi.12227>

Ramme, W. (1951). Zur systematik, faunistik und biologie der Orthopteren von Südost-Europa und Vorderasien. *Mitteilungen aus dem Zoologischen Museum in Berlin, 27*, 1-431.

Revell, L. J. (2012). phytools: An r package for phylogenetic comparative biology (and other things). *Methods in Ecology and Evolution, 3*, 217-223. <https://doi.org/10.1111/j.2041-210X.2011.00169.x>

Rochette, N. C., Rivera-Colón, A. G., & Catchen, J. M. (2019). stacks 2: Analytical methods for paired-end sequencing improve RADseq-based population genomics. *Molecular Ecology, 28*(21), 4737-4754. <https://doi.org/10.1111/mec.15253>

Rohlf, F. J. (1998). On applications of geometric morphometrics to studies of ontogeny and phylogeny. *Systematic Biology, 47*(1), 147-158. <https://doi.org/10.1080/106351598261094>

Ruxton, G. D., & Neuhauser, M. (2010). When should we use one-tailed hypothesis testing? *Methods in Ecology and Evolution, 1*(2), 114-117. <https://doi.org/10.1111/j.2041-210X.2010.00014.x>

Scudder, S. H. (1897). Revision of the Orthopteran group Melanopli (Acrididae) with special reference to North American forms. *Proceedings of the United States National Museum, 20*(1124), 1-421.

Solís-Lemus, C., Bastide, P., & Ane, C. (2017). phylonetworks: A package for phylogenetic networks. *Molecular Biology and Evolution, 34*(12), 3292-3298. <https://doi.org/10.1093/molbev/msx235>

Stamatakis, A. (2014). raxml version 8: a tool for phylogenetic analysis and post-analysis of large phylogenies. *Bioinformatics, 30*(9), 1312-1313. <https://doi.org/10.1093/bioinformatics/btu033>

Swofford, D. L. (2002). *paup∗. Phylogenetic analysis using parsimony (∗and other methods). Version 4*. Sunderland, MA: Sinauer Associates.

Uvarov, B. P. (1942). New and less known Southern Palaearctic Orthoptera. *Transactions of the American Entomological Society, 67*, 303-361.

Wang, I. J. (2013). Examining the full effects of landscape heterogeneity on spatial genetic variation: A multiple matrix regression approach for quantifying geographic and ecological isolation. *Evolution, 67*(12), 3403-3411. <https://doi.org/10.1111/evo.12134>

Wang, I. J. (2020). Topographic path analysis for modelling dispersal and functional connectivity: Calculating topographic distances using the *topoDistance* r package. *Methods in Ecology and Evolution, 11*(2), 265-272. <https://doi.org/10.1111/2041-210x.13317>

Warren, D. L., & Seifert, S. N. (2011). Ecological niche modeling in maxent: the importance of model complexity and the performance of model selection criteria. *Ecological Applications, 21*(2), 335-342. <https://doi.org/10.1890/10-1171.1>

Warren, D. L., Wright, A. N., Seifert, S. N., & Shaffer, H. B. (2014). Incorporating model complexity and spatial sampling bias into ecological niche models of climate change risks faced by 90 California vertebrate species of concern. *Diversity and Distributions, 20*(3), 334-343. <https://doi.org/10.1111/ddi.12160>

Willemse, F. (1971). The genus Oropodisma Uvarov, 1942, with the description of two new species (Orthoptera, Acridiidae, Catantopinae). *Publicaties van het Natuurhistorisch Genootschap in Limburg, 20*, 19-25.

Willemse, F. (1972a). Further records of the genus *Oropodisma* Uvarov, 1942 (Orthoptera, Acrididae, Catantopinae). *Publicaties van het Natuurhistorisch Genootschap in Limburg, 22*, 27-31.

Willemse, F. (1972b). Peripodisma tymphii, a new genus and species ot the tribe Podismi from Southeast Europe (Orthoptera, Acrididae, Catantopinae). *Publicaties van het Natuurhistorisch Genootschap in Limburg, 22*, 81-85.

Willemse, F. (1979). A new species and new distributional data of Oropodisma Uvarov, 1942 from Greece (Orthoptera, Acrididae). *Entomologische Berichten, Amsterdam, 39*, 108-111.

Willemse, F. (1984). Catalogue of the Orthoptera of Greece. *Fauna Graeciae, 1*(I-XII), 1-275.

Willemse, F., & Willemse, L. (2008). An annotated checklist of the Orthoptera-Saltatoria from Greece including an updated bibliography. *Articulata - Beiheft, 13*, 1-91.

Yannic, G., Ortego, J., Pellissier, L., Lecomte, N., Bernatchez, L., & Cote, S. D. (2018). Linking genetic and ecological differentiation in an ungulate with a circumpolar distribution. *Ecography, 41*(6), 922-937. <https://doi.org/10.1111/ecog.02995>
